# A precision medicine approach to interpret a GATA4 genetic variant in a paediatric patient with congenital heart disease

**DOI:** 10.1186/s40246-025-00907-6

**Published:** 2026-01-03

**Authors:** Catherine A. Forbes, Nicole C. Shaw, Kevin G. Chen, Mitchell Hedges, Teagan S. Er, Livia Hool, Michelle Ward, Cathryn Poulton, Gareth Baynam, Timo Lassmann, Vanessa S. Fear

**Affiliations:** 1 Translational Genetics, Precision Health, The Kids Research Institute Australia, Centre for Child Health Research, Perth Children’s Hospital, The University of Western Australia, Nedlands, WA 6009 Australia; 2 School of Human Sciences, The University of Western Australia, Crawley, WA Australia; 3 Victor Chang Cardiac Research Institute, Darlinghurst, NSW Australia; 4 Western Australian Register of Developmental Anomalies, King Edward Memorial Hospital, Subiaco, WA Australia; 5 Undiagnosed Diseases Program, Genetic Health, Subiaco, WA Australia; 6 Genetic Health of WA, King Edward Memorial Hospital, Subiaco, WA Australia

**Keywords:** GATA4, Induced pluripotent stem cells, CRISPR gene editing, Cardiac disease modelling, Congenital heart disease

## Abstract

**Background:**

Patients with congenital heart disease are identified in 1% of live births. Improved surgical intervention means many patients now survive to adulthood, the corollary of which is increased mortality in the over-65-year-old congenital heart disease (CHD) population. In the clinic, genetic sequencing increasingly identifies novel genetic variants in genes related to CHD. Traditional assays for interpreting novel genetic variants are often limited by gene-specificity, whereas animal models are cumbersome and may not accurately reflect human disease. This study investigates CRISPR gene editing in induced pluripotent stem cells and cardiomyocyte-directed differentiation as a human disease model to investigate novel genetic variants identified in association with CHD.

**Methods and results:**

We identified a GATA4 p.Arg284His genetic variant in a paediatric patient. This genetic variant was introduced into induced pluripotent stem cells (iPSCs) using CRISPR gene editing with homology-directed-repair. *GATA4* genetic variant and isogenic control iPSCs were selected and differentiated into cardiomyocytes. Expression of the GATA4 p.Arg284His variant resulted in altered calcium transients, indicative of CHD and consistent with the patient’s clinical phenotype. Transcriptomics revealed cellular pathway changes in cardiac development, calcium handling, and energy metabolism that contribute to disease aetiology, mechanism and identification of potential treatments.

**Conclusion:**

Directed differentiation of iPSCs harbouring the GATA4 p.Arg284His genetic variant recapitulated the CHD phenotype, indicated disease mechanisms, and pointed to potential sites for targeting with therapy. The study highlights the utility of transcriptomics for the functional interpretation of cardiac genetic variants and is an exemplar for precision medicine approaches for the investigation of CHD.

**Supplementary Information:**

The online version contains supplementary material available at 10.1186/s40246-025-00907-6.

## Introduction

Clinical genetic sequencing often reveals novel variants, or variants of uncertain significance (VUS), in patients with congenital heart disease (CHD). Diagnosis is complicated by the estimated 400 + genes that contribute to CHD pathogenesis [1] and their associated VUS [2]. Determining the functional consequences of these VUS requires investigation in a living cell system [3] to assess disease relevance. This study uses CRISPR Cas9 gene editing in iPSCs, cardiac disease modelling, and transcriptomics to investigate a novel GATA4 genetic variant identified in a paediatric patient with atrial septal defect (ASD).

CHD affects nearly 1% of all live births globally [4], and encompasses a range of heart defects including ventricular septal defect (VSD), atrial septal defect (ASD), and atrioventricular septal defect (AVSD). These are characterised by defects in the atrial and ventricular septum that allow blood flow between heart chambers. Other common clinical features include atrial or ventricular fibrillation, abnormal heart rhythm, shortness of breath, feeding problems, and failure to thrive. Today, surgical interventions have improved patient prognosis; however, as more children survive into adulthood, morbidity and mortality increase among ageing CHD patients [5].

GATA4 is a key transcription factor regulating cardiomyocyte differentiation and is expressed in all cardiac cells including progenitor cells, cardiomyocytes, endothelial cells, and fibroblasts [6]. The GATA4 protein contains two highly conserved carboxyl Zn finger domains that are key in the formation of transcription factor complexes to regulate DNA binding [7, 8]. GATA4 acts in concert with transcription factors and accessory proteins to regulate cardiomyocyte proliferation (Cyclin D2, CDK4, KLF13) [9–11], cardiogenesis (GATA6, MEF2A/C/D, TBX5, HAND2, NKX2.5, YY1, SMAD4, RXRa) [12], valve and septal formation (GATA5, FOG2) [13, 14], and cardiac hypertrophy (GATA6, MEF2C, HEY2, NFAT3, NKX2.5, P300, among others) [14–16].

Mutations in the *GATA4* gene have been reported in a wide range of CHD phenotypes, including atrial fibrillation, septal defects, outflow tract defects, pulmonary valve stenosis, misplaced aorta, and right ventricular hypertrophy [6].

In this study, we investigate the functional consequences of a novel GATA4 c.851G > A p.Arg284His genetic variant, located in the GATA4 carboxy Zn finger domain, identified in a paediatric patient with CHD. We introduced the variant into iPSCs via CRISPR single-base editing, and evaluated cardiomyocyte differentiation for changes in cell structure, pathways, and functional activity. Cellular changes mirrored the patient’s CHD phenotype, and transcriptomics identified key pathways underlying disease aetiology and mechanism that could be targeted therapeutically. In addition, as the variant is located within the carboxyl zinc finger domain of the protein, our data also provide evidence that this site is critical to DNA binding, cardiomyocyte differentiation and embryonic development.

## Methods

### Patient recruitment

Patient recruitment was initiated by a genetic counsellor at Genetic Health Western Australia, followed by written informed consent. This study adheres to the Declaration of Helsinki and the NHMRC National Statement on Ethical Conduct in Human Ethics Research and was approved by the Child and Adolescent Health Services, Human Research Ethics Committee, RGS0166.

### Patient genome sequencing and human phenotype ontology

Target enrichment was carried out using TWIST Exome Panel (101952) and TWIST EF Library Prep Kit (101901) on a prenatal amniocentesis sample from the patient (PathWest, Western Australia). Massively parallel whole exome sequencing with bioinformatically targeted analysis of the Disorders of Sex Differentiation gene panel was performed. Primary analyses were performed on an Illumina NextSeq550 with High Output Kit v2.5 (20024908). Secondary analyses were performed using DRAGEN Enrichment v3.6.3 with GRCh38 alignment. Tertiary analyses were completed using Alissa Interpret v5.3 (Agilent Technologies) and associated annotation sources. The variant was classified as a VUS according to ACMG guidelines (PathWest, Australia; [17]).

### iPS cell culture

KOLF2-C1 (KOLF2) cells were grown in 6-well plates coated with Matrigel^®^ hESC-Qualified Matrix, LDEV-free (Corning^®^) maintained in TeSR-E8 media (STEMCELL Technologies), and media changed daily. Cells were split with Gentle Cell Dissociation Reagent (STEMCELL Technologies) [18]. Cell cryopreservation was in KnockOut™ Serum Replacement medium (Gibco™) supplemented with 10% DMSO. All cultures were maintained at 37 °C humidified 5% CO_2_ incubator, unless otherwise stated, and were routinely tested for mycoplasma.

### GATA4 CRISPR gene editing

KOLF2 cells were CRISPR gene edited as previously described [18–22]. In brief, cells were grown to 30–50% confluence, dissociated with Gentle Cell Dissociation Reagent and 1 × 10^5^ cells in 400 µl TeSR-E8 with 10 µM Y-27632 (STEMCELL technologies) aliquoted in 24-well plates. The GATA4 p.Arg284His_HDR ssDNA (^5′Alt-R-HDR1^ tgtgccaactgccagaccaccaccaccacgctgtggcgccacaatgcggagggcgagcctgtgtgcaatgcctgcggcctcta^Alt-R-HDR2 3′^), and GATA4 p.Arg284His crRNA (5′ CACGCTGTGGCGCCGCAATG 3′, PAM: CGG; GATA4 NM_002052.4), were purchased from Integrated DNA Technology (IDT). First the gRNA was generated in Duplex Buffer (IDT, USA) with 1µM crRNA (IDT), 1µM tracrRNA-ATTO550 (IDT), heated to 95 °C for 5 min and cooled to RT. CRISPR ribonucleoprotein (RNP) complexes were formed with 63nM gRNA and 63nM high-fidelity Cas9 protein (IDT) in OPTIMEM, followed by addition of 63 nM HDR. RNPs were transfected into KOLF2 cells with STEM Lipofectamine (Life Technologies), cultures were treated with 30µM ALT-R HDR enhancer and incubated for 2 days at 32 °C with a media change to remove Alt-R HDR Enhancer at 24 h. Cells were cultured a further 7–14 days prior to cell freeze-down and genomic DNA (gDNA) extraction (DNeasy Blood & Tissue Kit, QIAGEN). Percentage frequency gene editing in gDNA was determined by amplicon sequencing [18–23]. Transfected cells were single-cell cloned by plate picking, and a second round of amplicon sequencing performed on gDNA to determine clonal cell lines.

The mutalyzer chromosomal descriptions for the introduced GATA4 p.Arg284His variant are GRCh38 NC_000008.11(NM_002052.5): c.851G > A or NC_000008.11:g.11750178G > A; and protein prediction NM_002052.5(NP_002043.2):p.(Arg284His).

### Amplicon sequencing for detection of GATA4 gene editing

Next-generation amplicon sequencing was carried out on the MiniSeq Sequencing System (Illumina©). In brief, a 250bp *GATA4*_HDR site PCR product was amplified, from gDNA, with *GATA4* pAMPF1 (^5′^ACACTCTTTCCCTACACGACGCTCTTCCGATCTcaccttttacttggacatgaagc^3′^) and *GATA4* pAMPR1 (^5′^GTGACTGGAGTTCAGACGTGTGCTCTTCCGATCTgtacaaaggaagaagacaaggga^3′^) [23] for 150 bp, paired end, > 10,000 reads (MiniSeq, Illumina, Australia) and reads aligned to the HDR or WT amplicon with CRISPResso2 software [24].

FASTQ file amplicon reads were aligned to sequences for GATA4 WT: ^5′^caccttttacttggacatgaagcatttgtttcctgtcttgcagtccgcctcccgccgagtgggcctctcctgtgccaactgccagaccaccaccaccacgctgtggcgccgcaatgcggagggcgagcctgtgtgcaatgcctgcggcctctacatgaagctccacggggtacgtgggtcctgcgcccatgcggcatccttgccttctgatgcccatctctcagtcctcccttgtcttcttcctttgtac^3′^; and GATA4 p.Arg284His: ^5′^ caccttttacttggacatgaagcatttgtttcctgtcttgcagtccgcctcccgccgagtgggcctctcctgtgccaactgccagaccaccaccaccacgctgtggcgccgcaatgcggagggcgagcctgtgtgcaatgcctgcggcctctacatgaagctccacggggtacgtgggtcctgcgcccatgcggcatccttgccttctgatgcccatctctcagtcctcccttgtcttcttcctttgtac^3′^. Code in Supplemental Experimental Procedures_Paper Analysis_GATA4 VUS, GATA4 VUS CRISPResso Script.

### Off-target analysis and karyotyping

The top six off-target CRISPR crRNA gene cut sites for the GATA4_p.Arg284His crRNA (5′ AGACCACCACCACCACGCTG 3′, PAM: TGG; GATA4 NM_002052.4) were assessed with PCR amplification (Suppl Table 1) followed by forward and reverse Sanger sequencing for all cell clones (AGRF, WA). Karyotyping was performed on gDNA extracted from each iPSC line using the hPSC Genetic Analysis Kit (STEMCell Technologies).

### Cardiomyocyte differentiation

Both variant and control KOLF2 iPSCs, were differentiated using the STEMdiff Ventricular or Atrial Cardiomyocyte Differentiation Kits (STEMCELL, Australia) and subsequently maintained in the corresponding Maintenance Kit. At the indicated timepoint for iPSCs and cardiomyocytes, 5 × 10^5^ cells were collected, fixed/permeabilized according to Transcription factor staining buffer set (eBioscience, US), LIVEDEAD Fixable Viability Dye eFluor™ 780 stained (eBioscience, US), and assessed for stem cell marker expression: OCT3/4 (OCT3/4-AF488, 1:20, clone 40/Oct-3 RUO, BD Pharmingen) and NANOG (NANOG-BV421, 1:20, clone 16H3A48, BioLegend); and/or cardiomyocyte marker expression: GATA4 (GATA4-PE, 1:20, clone L97-56, BD Pharmingen), TNNT2 (cTnT-AF647, 1:20, clone 13 − 11, RUO, BD Pharmingen). Samples were collected using a BD LSRFortessa flow cytometer with BD FACSDiva™ software (BD Biosciences), and analysed with FlowJo software (TreeStar Inc, Ashlan, OR, USA). In addition, cells were visualised by light microscopy, and representative video captured of beating cardiomyocytes. Beating cardiomyocytes were scored [1–5] according to the percentage of beating cells: 1, *≥* 5%; 2, *≥* 10%; 3, *≥* 20%; 4, 50–80%; and 5, > 80%. Statistical analysis of data was performed using GraphPad Prism Version 10.1 software (GraphPad Software Inc., La Jolla, CA, USA).

For immunofluorescence microscopy, iPSCs were cultured on Matrigel-coated chamber slides (ibidi), differentiated into cardiomyocytes, fixed with 3.7% formaldehyde (Sigma-Aldrich) for 20 min at room temperature and stored at 4 °C in DPBS. Next, cells were permeabilised 15 min in 0.1% Triton-X-100 (Sigma-Aldrich), blocked with Intercept^®^ Blocking Buffer (LI-COR) for 1 h room temperature, and incubated at 4 °C overnight with rabbit anti-GATA4 antibody (1:400; D3A3M, Cell Signalling Technology). Cells were washed with 0.05% Tween-20 (Sigma-Aldrich), incubated with Alexa-Fluor 488-conjugated anti-rabbit antibody (1:1000; Invitrogen) for 1 h at room temperature, and washed prior to NucBlue staining (Invitrogen), prior to visualisation on a Nikon C2 + inverted confocal microscope. Images were captured and processed using NIS-Elements software (v.5.21.00) and Image J FIJI. To visualise sarcomeric proteins in cardiomyocytes, primary antibodies to α-actinin2 (rabbit, clone 7H1L69, Invitrogen), cTnT (mouse, clone 13 − 11, NeoMarkers) and myosin binding protein C (mouse, clone E-7, Santa Cruz) were used in combination with anti-rabbit-AF488 and anti-mouse-AF568 secondary antibodies and imaged using a THUNDER imaging system (Leica). Z-stacks were captured and Leica technology computational clearing applied to minimise noise prior to obtaining a maximum intensity projection image using AIVIA software (Version 15.0).

### Measurement of calcium transients

The assessment of intracellular calcium was performed as previously described [21]. Cells were incubated with 5 µM Fluo-4 (Life Technologies) in StemDiff media (StemCell) for 20 min, followed by a 20-minute de-esterification step in HEPES-Buffered Solution (HBS) containing (in mM): 5.3 KCl, 0.4 MgSO_4_.7H_2_O, 139 NaCl, 1.8 CaCl_2_, 5.6 Na_2_HPO_4_.2H_2_O, 5 glucose and 20 HEPES (pH = 7.4 at 37 °C), not supplemented with Fluo-4. Fluo-4 fluorescence was then recorded in HBS not containing Fluo-4 at 37 °C using fast acquisition at 10 ms intervals, with a x40 objective using a Zyla 5.5 sCMOS camera attached to an inverted Nikon Eclipse Ti2 microscope (ex 480 nm, em 535 nm). NIS elements AR was used to quantify the signal by manually tracing myocytes. An equivalent region not containing cells was used as background and subtracted. The signal for each cell (F) was normalised to basal signal (F_0_), yielding F/F_0_, therefore basal signal approximated 1.0. The frequency of spontaneous calcium transients was calculated as the number of transients over the total length of recording (4 s), full width of half maximum (FWHM) was calculated by fitting a Lorentzian function to the first calcium transient on each recording using GraphPad Prism10.1.2 software [25–27].

### RNA sequencing

Cultured iPSCs or cardiomyocytes were collected for RNA extraction (RNeasy Minikit, with DNase on column treatment; Qiagen). RNA integrity was determined on the bioanalyser (Australian Genomics Research Facility (AGRF), Perth, Western Australia). RNA libraries were prepared with the SureSelect Strand-Specific XT HS2 Poly-A kit and sequenced at Genomics WA (Perth, Australia) on an Illumina NovaSeq 6000 platform (paired-end, 100 bp. ~30 million reads per sample).

### Data processing and alignment

UMItools [28] was used to de-duplicate before alignment. All reads were aligned to the human genome (build GRCh38), using a modified version of the ENCODE ‘rna-seq-pipeline’ (https://github.com/ENCODE-DCC/rna-seq-pipeline), via the Cromwell wrapper software ‘caper’ (https://github.com/ENCODE-DCC/caper). Within the pipeline, reads were aligned to GRCh38 using the STAR aligner (v2.5.1b) [29] and known transcripts were quantified using Kallisto (v0.44.0) [30]. We performed basic QC analysis of mapped reads using SAMStat [31].

### Differential gene expression analysis

Estimated gene abundances were imported into R (v4.4.3) (https://www.R-project.org/) using the tximport (v1.3.2) package [32], and a standard RNAseq analysis was applied using edgeR, limma and voom as previously described [22].

### Biological interpretation of sample differences

GSEA functions within the clusterProfiler (v4.12.2) [33] package were used to perform preranked gene set enrichment analysis. Genes were ranked based on their log fold changes, with enrichment analyses performed against gene sets within Gene Ontology, KEGG, and DisGeNET. The full analysis script is reported in Supplemental Experimental Procedures_Paper Analysis VUS, GATA4 VUS2 – supplementary code.

## Results

### Patient clinical information

**Fig. 1:**
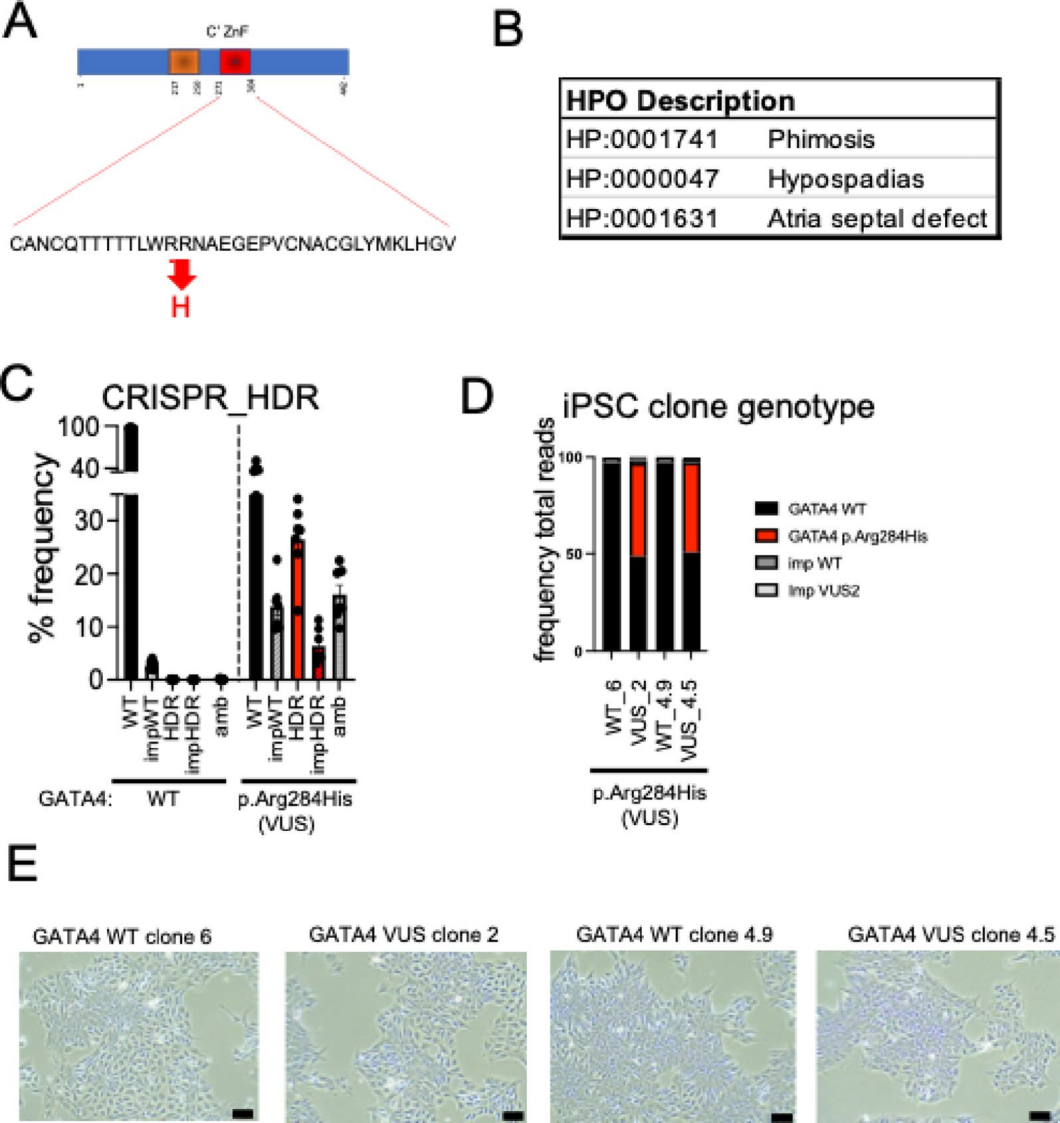
GATA4 VUS genetic variant and stem-cell gene editing. **A** Protein structure showing the patient mutation in the carboxyl zinc finger domain. **B** Patient Human Phenotype Ontology terms as described by physician. **C** CRISPR gene editing efficiency for single nucleotide variant introduction in KOLF2C iPSCs sequencing reads aligned to GATA4_WT and GATA4 VUS amplicon sequence. WT, sequencing reads aligned to WT sequence. Imp WT, imperfect sequencing reads aligned to WT with other mutations. HDR, reads aligned to the VUS sequence. Amb, sequencing reads that align to the GATA4 amplicon that have deleted the target site. **D** Bar graph showing variant representation in genomic DNA from healthy (WT) and heterozygous GATA4 p.Arg284His (VUS) clones, with clone identity. **E** Light microscopy images demonstrating normal stem-cell morphology. Scale bar, 100 micron

The GATA4 p.Arg284His VUS is located within the carboxyl zinc finger domain of the protein, critical for complex formation with co-factors and subsequent DNA binding (Fig. 1A). We hypothesized that the genetic variant would disrupt transcriptional regulation leading to perturbations in cardiomyocyte differentiation and function.

The proband presented in pregnancy with ambiguous genitalia, and a post-natal echocardiogram revealed an ASD. The Human Phenotype Ontology (HPO) terms provided by the patient’s geneticist clinician indicated phimosis (HP:0001741), hypospadias (HP:000047), and atrial septal defect (HP:0001631) (Fig. 1B). At age 1 year the proband underwent hypospadias repair and at age 3 years underwent closure of ASD. At last review (4 years old), the proband height was on the 75th percentile, weight and head circumference on the 50th percentile. No dysmorphic features were reported, systemic examination was within normal limits, and developmental was appropriate.

Whole exome sequencing identified a heterozygous missense variant in the *GATA4* gene c.851G > A p.Arg284His classified as a VUS. Variants of uncertain significance considered risk factors of unknown penetrance and carrier status were not reported. The variant classification was according to ACMG guidelines [17], citing a highly conserved Arginine residue which is located in the Zn finger domain of the protein indicative of moderate support for pathogenicity (PM1); the variant was not found in the population variant database gnomAD as moderate support (PM2); and, multiple in silico algorithms in support of possible pathogenicity (PP3). Therefore, there were two moderate (PM1, PM2) and one supporting (PP3) criteria and according to ACMG requirements the variant was classified as a VUS. Notably, the variant has been reported in two families with ASD, with variable expression of the associated phenotype [34, 35].

CNVs were normal for the targeted Inherited disorders gene panel (AKR1C2, AKR1C4, AMH, AMHR2, ANOS1, AR, ARX, ATF3, ATRX, VMP15, CDKN1C, CHD7, CYP11A1, CYP11B1, CYP17A1, CYP19A1, DHCR7, DHH, DUXP6, FEXF1, FGF17, FGF8, FGFR1, FLRT3, FSHB, FSHR, GATA4, GNRH1,GNRHR, HESX1, HS6ST1, HSD17B3, HSD17B4, HSD3B2, IL17RD, INSL3, KISS1R, LEP, LEPR, LHB, LHCGR, LHX3, MAMLD1, MAP3K1, MKKS, NR0B1, NR3C1, NR5A1, PCSK1,POR, PROK2, PROP1, RSPO1,SEMA3A, SOX9, SRD5A2, SRY, STAR, TAC3, TACR3, TOE1, TSPYL1, WDR11, WNT4, WT1, ZFPM2), variants in regions of low coverage may not have been identified.

We determined to investigate how the introduction of the genetic variant in iPSCs would affect cardiomyocyte differentiation and development and provide in vivo functional studies that may support a damaging effect of the gene or gene product (PS3). According to ACMG requirements the provision of PS3 evidence means the VUS would meet likely pathogenic criteria (1 strong, 2 moderate, 1 supporting).

### GATA4 genetic variant introduction in iPSCs

**Fig. 2:**
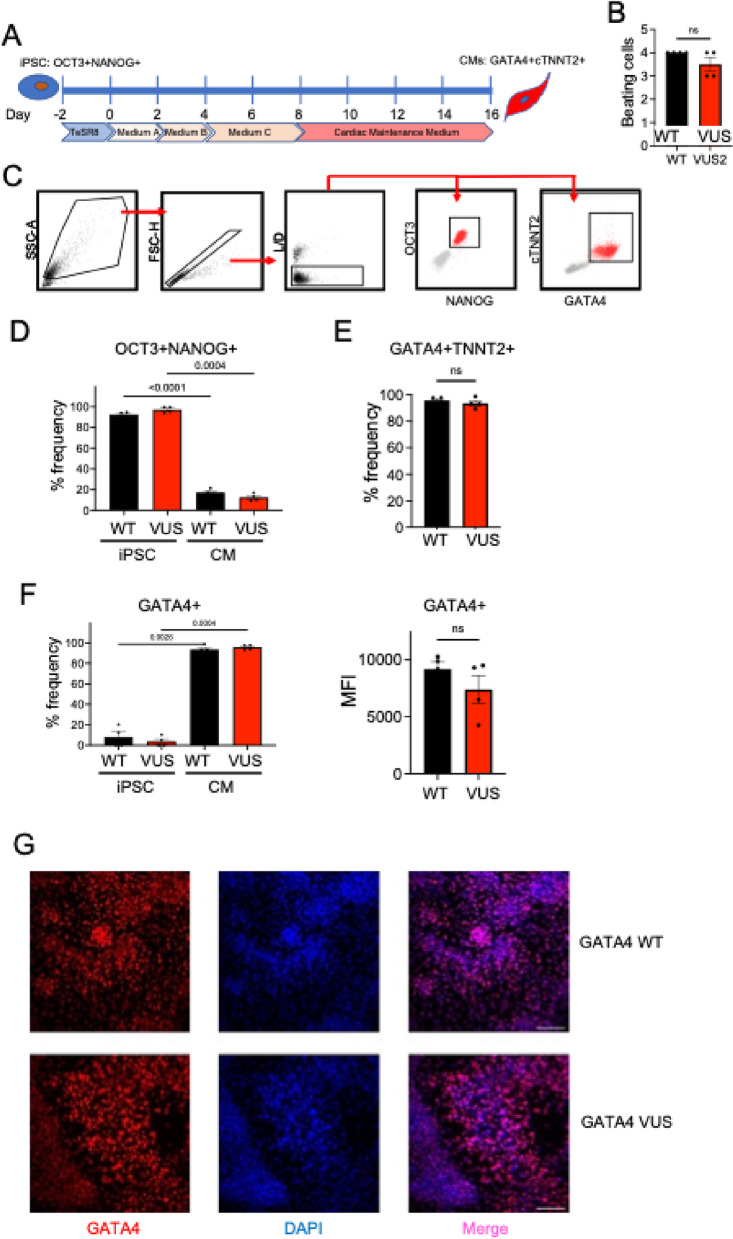
GATA4 WT and GATA4 VUS iPSC cardiac disease modelling. **A** iPSC cardiomyocyte differentiation schematic. **B** Beating score for cardiomyocytes. **C** Flow cytometry gating. Cells were gated on forward and side scatter, single cells, and cell viability; with subsequent gating for OCT3 and NANOG expression, or TNNT2 and GATA4 expression. **D** Bar plots indicate OCT3^+^NANOG^+^ percentage frequency expression in GATA4 WT and GATA4 VUS cells at day 0 (iPSCs) and day 16 (cardiomyocytes, CM). **E** Bar plots indicate expression percentage frequency GATA4^+^TNNT2^+^ in cardiomyocytes. **F** Bar plots indicate GATA4% frequency expression in iPSCs and cardiomyocytes, and GATA4 mean fluorescent intensity (MFI) in cardiomyocytes. **G** Fluorescent immunohistochemistry indicates that GATA4 WT and GATA4 VUS protein localizes to the nucleus. MFI, mean fluorescent intensity. Scale bar, 100 micron. Data represents two experiments with paired WT and VUS clones (GATA4_WT clone 6 and GATA4_VUS clone 2; GATA4_WT clone 4.9 and GATA4_VUS clone 4.5). *p *≤* 0.05, two-way ANOVA, with Bonferonni’s correction for multiple testing)

CRISPR/Cas9-mediated homology-directed repair introduced the *GATA4* c.851G > A; p.Arg284His variant into KOLF2 iPSCs. Amplicon sequencing [21, 24] determined incorporation of the single base edit at 26.5 ± 2.6% (Mean ± SE; Fig. 1B). Single cell cloning and genotyping using *GATA4* targeted amplicon sequencing on gDNA (minimum 35,000 reads/cell) identified two heterozygous *GATA4* c.851G > A; p.Arg284His clones (Fig. 1C) and matched isogenic controls. All iPSCs maintained normal stem cell morphology (Fig. 1D), genomic DNA integrity at off-target crRNA sites (Suppl Fig. 1A), normal karyotype (Suppl Fig. 1B), and expressed stem cell markers OCT3 and NANOG (Fig. 2D).

### GATA4 genetic variant cardiomyocyte differentiation and marker expression

**Fig. 3:**
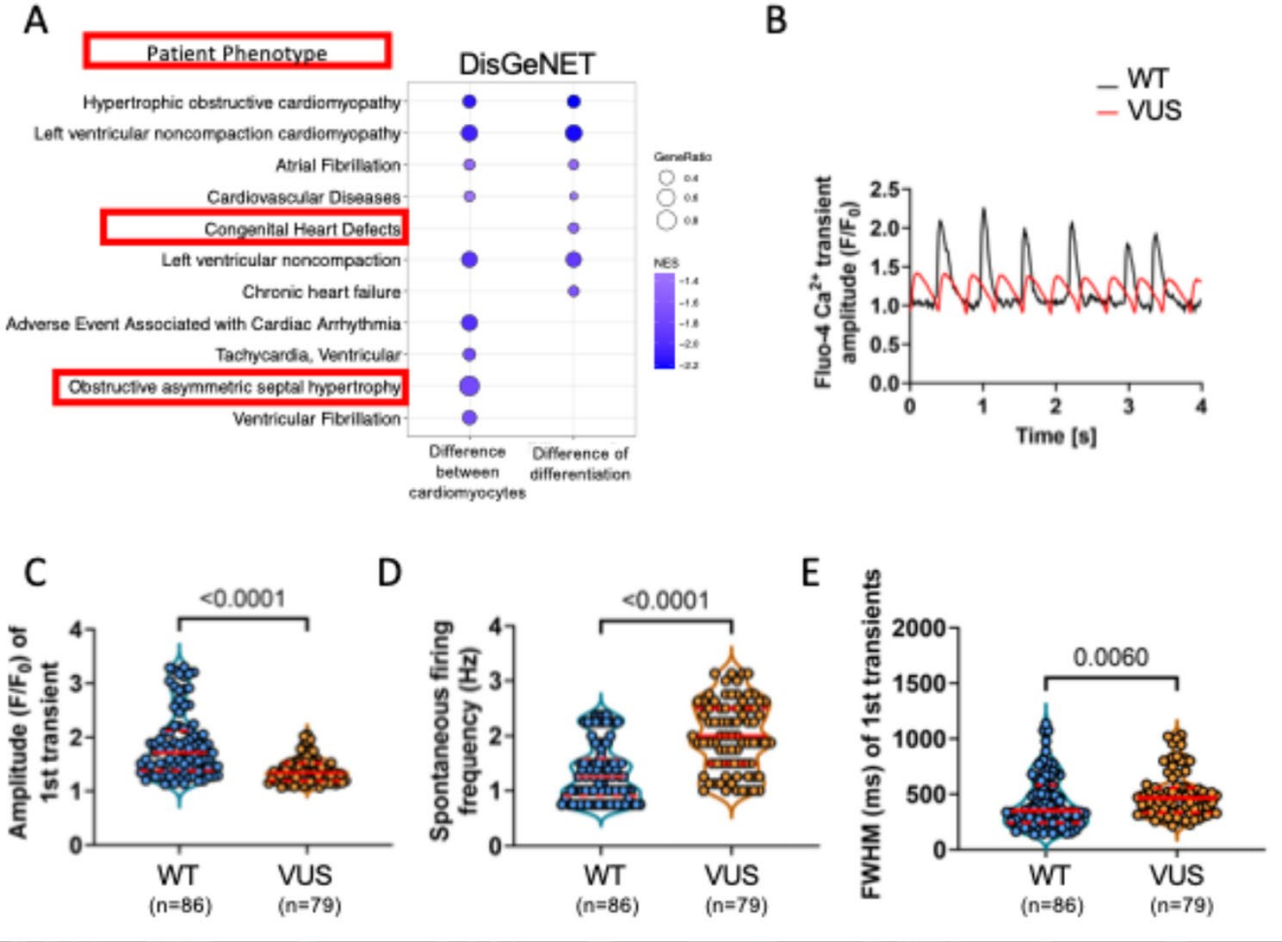
Changes in GATA4 VUS cardiomyocytes and cardiac differentiation reflect patient disease phenotype. GATA4 WT and GATA4 VUS iPSCs were stimulated to form cardiomyocytes. **A** Bubble plot indicates GSEA pathway changes using the DisGeNET data set, for the difference between ventricular cardiomyocytes, and the differences in differentiation. **B** Alterations in calcium transients in GATA4 WT and GATA4 VUS atrial cardiomyocytes. **C–E** Graphs indicate changes in Amplitude, spontaneous firing, and FWHM for GATA4 WT and GATA4 VUS cardiomyocytes, as indicated. (Two independent experiments, Mann-Whitney test)

The *GATA4* c.851G > A; p.Arg284His genetic variant (GATA4 VUS) iPSCs and matched isogenic (GATA4 WT) iPSCs were differentiated into functional, beating cardiomyocytes (Fig. 2A, B, Supplementary videos). In flow cytometry analysis, there were no significant differences in the expression of stem (OCT3, NANOG) or cardiac (GATA4, TNNT2) cell markers (Fig. 2D, E) between GATA4 WT and GATA4 VUS cells. GATA4 protein expression (Fig. 3F), and protein localisation to the nucleus (Fig. 2G), were similar in both the GATA4 WT and GATA4 VUS cardiomyocytes.

Transcriptomics was used to determine changes in cardiomyocyte differentiation. Principal component analysis (PCA) clearly separated the iPSCs from cardiac cells (Suppl Fig. 2A). In the comparison of cardiomyocytes to iPSCs there were 11,174 significant differentially expressed genes (DEGs; Benjamini-Hochberg adjusted p-value < 0.05) in GATA4 WT; and 11,175 in GATA4 VUS (Suppl Tables 2 and Suppl Table 3). This included significant upregulation of the cardiac genes (ACTC1, ACTN2, GATA4, MYBPC3, MYH6, MYH7, MYL7, and TNNT2; Suppl Fig. 2C), and down regulation of stem cell markers POU5F1 (OCT3/4), NANOG and SOX2 in both healthy and genetic variant cells (Suppl Fig. 2B).

Gene set enrichment analysis (GSEA) was performed using the Gene Ontology Biological Processes (GO-BP) database, identifying changes in cellular pathways during GATA4 WT and GATA4 VUS cardiomyocyte differentiation. Within the top ten significantly up-regulated pathway gene sets were: myofibril assembly; cardiocyte differentiation; cardiac muscle cell differentiation; cardiac muscle development; and cardiac muscle tissue development (Suppl Tables 4 and 5).

These data indicate successful differentiation of GATA4 WT and GATA4 VUS iPSC to cardiomyocytes.

### Phenotypic signature of patient-specific disease

To assess the disease relevance of the model, we compared GATA4 VUS and GATA4 WT transcriptomes by performing GSEA against the DisGeNET database. As GATA4 is linked to cardiac cell development, we compared cardiomyocytes [CM_VUS_ – CM_WT_], as well as difference in differentiation ([CM_VUS_ – iPSC_VUS_] - [CM_WT_ – iPSC_WT_]).

Comparing the cardiomyocyte transcriptomes highlighted the pathway term “Obstructive asymmetric septal hypertrophy,” whereas the differentiation comparison revealed “Congenital Heart Defects” (Fig. 3A). Other enriched pathway terms relevant to cardiomyocyte dysfunction in CHD included: adverse event associated with cardiac arrythmia; ventricular fibrillation; ventricular tachycardia; left ventricular noncompaction cardiomyopathy; and atrial fibrillation (Fig. 3A; Suppl Tables 6 and 7).

### GATA4 VUS disrupts calcium handling

Calcium flux assays were next performed to investigate changes in calcium sequestration in GATA4 VUS and GATA4 WT cardiomyocytes. The GATA4 VUS cardiomyocytes demonstrated significantly increased spontaneous firing frequency, altered amplitude of the first transient, and increased FWHM (Fig. 3B, C). The altered rate and shape of the calcium transients indicated altered calcium cycling and excitation contract coupling. Calcium transients were regular but increased in frequency in the GATA4_VUS cells indicating faster excitation. The mutant cells also exhibited transients with reduced amplitudes and altered widths reflecting alterations in calcium release and uptake from the sarcoplasmic reticulum. The rate of beating was faster but regular in all GATA4_VUS cells suggesting a tachycardia.

These findings indicate decreased calcium sensitivity and contractile strength in the GATA4 VUS cardiomyocytes. Similarly, in previous studies, expression of GATA4 genetic variants in cardiomyocytes altered calcium handling [21].

The changes in calcium handling in the GATA4 VUS cardiomyocytes were consistent with the patient phenotype of ASD, and tachycardia in CHD.

### Patient specific pathway changes and disease mechanism

**Fig. 4:**
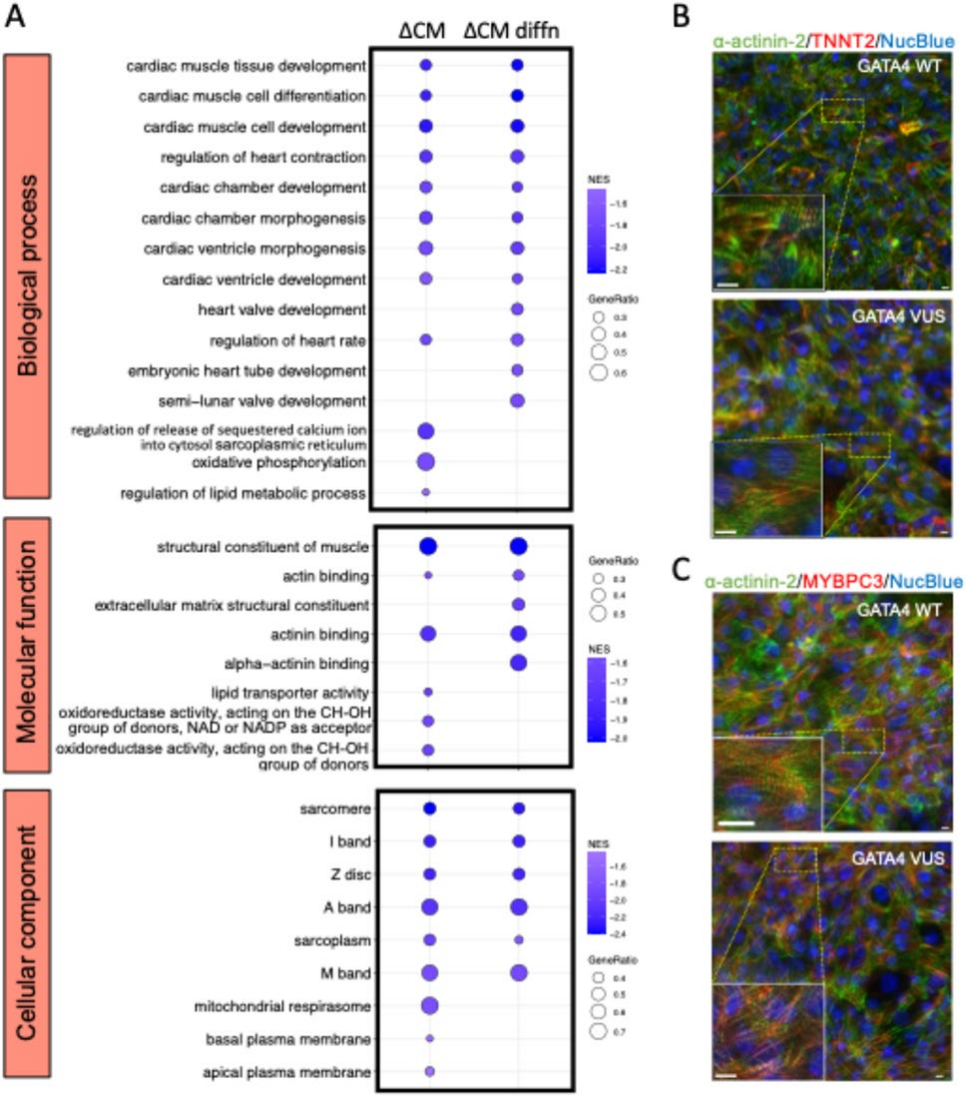
Pathway changes due to GATA4 VUS genetic variant expression in cardiomyocyte function and differentiation. **A** Transcriptomics with GSEA and Gene Ontology database comparing GATA4 VUS to GATA4 WT for the differences between cardiomyocytes (∆CM), and the difference in differentiation (∆diffn) in GO databases -Biological Processes, Molecular Function, and Cellular Component. (*n* = 4 experiments) **B** TNNT2 and a-actinin-2 staining of cardiomyocytes. Scale bar, 10 µM. **C** MYBPC3 and a-actinin-2 staining of cardiomyocytes. Scale bar, 10 micron

**Fig. 5:**
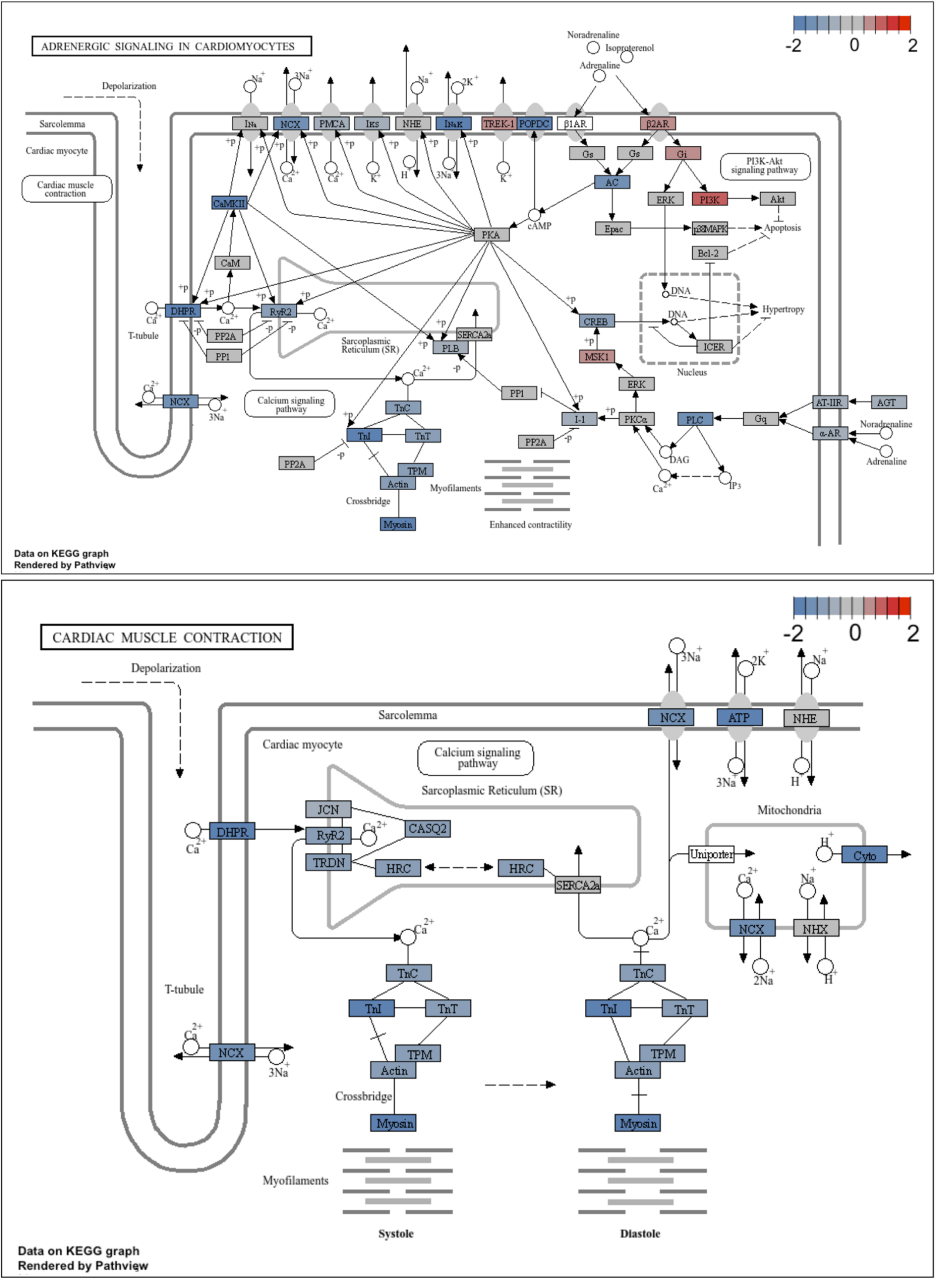
Changes in calcium pathways in GATA4 VUS genetic variant cardiomyocytes. Transcriptomics analysis with GSEA and KEGG database comparing GATA4 VUS to GATA4 WT for the differences between cardiomyocytes, pathway changes in Adrenergic signalling in cardiomyocytes and Cardiac muscle contraction, as indicated

To investigate disease mechanism, we compared GATA4 VUS to GATA4 WT cells to identify the DEGs (Suppl Table 8; Suppl Fig. 3) and performed GSEA against Gene Ontology in the subontologies - Biological Processes (GO-BP), Molecular Function (GO-MF), Cellular Component (GO-CC), and KEGG (Fig. 4A; Suppl Fig. 4; Fig. 5; and Suppl Tables 9–15) to identify changes in cellular pathway.

There were 49 significant DEGs (adj. p *≤* 0.05) in the comparison of GATA4 VUS to GATA4 WT cardiomyocytes (Suppl Fig. 3; Suppl Table 8), including the upregulation of six genes (PTGFR, BNC1, PRAG1, IQGAP1, TXNDC5, RAP2C), five of which were linked to cardiac dysfunction. PTGFR is implicated in hypertrophic cardiomyocyte growth, vascular remodelling, and heart failure [36]. PRAG1 is linked to cardiovascular disease [37], TXNDC5 promotes ECM formation in cardiac fibrosis [38], and IQGAP1 is a scaffold protein for the RAS/MAPK signalling cascade [39] and is essential for heart development in zebrafish [40]. RAP2C is a RAS/MAPK signalling protein, and mutations in this gene are a cause of RASopathy syndrome which often presents with cardiac anomalies [41]. Interestingly, whilst BNC1 is not associated with CHD or ASD, it has been linked to hypospadias [42], one of the HPO terms listed for the patient.

In downregulated DEGs, many genes were relevant to cardiac development and CHD including: HOPX which acts in concert with GATA4 and NKX2.5 during cardiomyocyte differentiation [43]; RCAN2 a regulator of calcineurin-NFAT signalling pathway required for cardiac development [44]; CORIN which is essential for heart morphogenesis [45]; NPPA which encodes atrial natriuretic peptide and is a marker for cardiac chamber specification [46]; DGCR6, in the cardiac neural crest, associated with outflow heart defects [47]; as well as CACNA1B [48], TECRL [49] and TRDN [50] genes associated with CHD arrythmia.

Next, changes in cellular pathways were investigated using GSEA against GO subontologies, comparing GATA4 VUS to GATA4 WT for differences in cardiomyocytes and the difference in differentiation (Fig. 4A; Suppl Tables 9–14). Significantly dysregulated cardiac pathways included: cardiac muscle cell differentiation; cardiac muscle tissue development; cardiac chamber development; cardiac chamber morphogenesis; cardiac ventricle development; cardiac ventricle morphogenesis; and regulation of heart contraction. Specific to the difference in differentiation were the pathway terms: semi-lunar valve development; heart valve development; and embryonic heart tube development. Notably, transcriptomics identified significant downregulation of the gene set pathway term ‘regulation of release of sequestered calcium ion in the cytosol sarcoplasmic reticulum’ in GATA4 VUS compared to GATA WT. A similar pattern was observed in other gene sets involving the formation of the sarcomere complex. To further investigate changes in sarcomere complex formation we performed immunostaining with a-actinin-2, TNNT2 and MYBPC3 in GATA4 WT and GATA4 VUS cardiomyocytes (Fig. 4B and C). Staining determined similar sarcomere structure in both the GATA4 WT and GATA4 VUS cardiomyocytes.

Other enriched GO gene set pathway terms when comparing GATA4 VUS to GATA4 WT related to energy metabolism, and included: oxidative phosphorylation, regulation of lipid metabolic process, lipid transporter activity, oxidoreductase activity, and mitochondrial respirasome.

Finally, GSEA of KEGG pathways for the difference in differentiation (Suppl Table 15) revealed downregulated pathways in GATA4 VUS including: Adrenergic signalling in cardiomyocytes, and cardiac muscle contraction, relevant to the cellular pathways for reduced calcium sensitivity and contraction (Fig. 5), respectively. In addition, GATA4 VUS cardiomyocytes showed down-regulation of PPAR signalling pathway, and a decrease in oxidative phosphorylation pathway (Suppl Fig. 4).

Overall, compared to GATA4 WT, the GATA4 VUS cardiomyocytes revealed changes in molecular and cellular pathways in cardiac development, calcium signalling, muscle contraction, and energy metabolism.

## Discussion

The study utilises CRISPR gene editing [18–22] and iPSC cardiomyocyte directed differentiation to assess a novel GATA4 (p.Arg284His; VUS) genetic variant identified in a paediatric patient with CHD. GATA4 VUS cardiomyocyte differentiation provided a model of disease that was in keeping with CHD and the patients clinically assessed disease phenotype. Importantly, the model pinpointed key cellular and molecular changes relevant to CHD including delayed differentiation, altered calcium handling, and decreased energy metabolism. Furthermore, disease aetiology and key cellular pathways were identified that may provide avenues for targeted treatment in both acute and chronic CHD to restore healthy cell function.

The precision medicine approach in this study utilised single base CRISPR gene editing with iPSC directed cardiomyocyte differentiation, to create a pre-clinical disease model that recapitulated the patient-specific disease. These striking findings may be attributed to the use of a human disease model and a disease-relevant cell type. Furthermore, comparison of the GATA4 VUS with isogenic control cells allowed for the direct attribution of changes in cell function due to the introduced genetic variant. This enabled key changes to be pinpointed at a cellular level and supported discovery of disease mechanisms. In keeping with findings by others, we postulate that key mechanistic insights were identified through the utilisation of human and cardiac disease models that may not always be accessible in traditional animal, or other, disease models [18, 19, 21, 22].

In the GATA4 VUS cardiomyocytes there were changes in cell function including decreased calcium transients with slower rise and recovery rates, and pathway changes indicating dysfunction in both calcium handling in the sarcoplasmic reticulum, and formation of the sarcomere. Findings of altered calcium transients are consistent with previous findings indicating cardiac dysfunction in CHD [51]. Interestingly, down-regulation of cardiac sarcomere proteins has been observed in infants with atrial septal defect studies [52], whilst other studies indicate that changes in GATA4 expression impact sarcomere formation [53]. These findings likely reflect the central function of the GATA4 transcription factor in cardiogenesis.

During heart development, the embryonic myocardium is primarily reliant on anaerobic glycolysis. However, as cardiac development progresses the adult cardiomyocytes transition to oxidative mitochondrial metabolism [54] to provide the increased energy requirements for cardiomyocyte differentiation and function [55]. Similarly, during in vitro differentiation of iPSC-derived cardiomyocytes, a switch in energy metabolism is observed during cell maturation, and disruption in this process has been shown to impact cardiomyocyte sarcomere formation and cell contractility [56]. In this study, GATA4 VUS iPSC-derived cardiomyocytes were delayed in their differentiation with a decrease in the transition to oxidative phosphorylation metabolism. Potentially, these changes may reflect a physiological state where the cardiomyocytes are inherently less mature, and/or that the lack of transition to oxidative phosphorylation impedes cardiac cell maturation.

Research findings by others, investigating cellular changes in early cardiac cell development have shown that the transcription factor complex PPAR/RXRa regulates gene expression [57]. In this study, the GATA4 VUS iPSC-derived cardiomyocytes downregulated the PPAR pathway and had reduced *RXRG* gene expression. Research investigating mechanisms of human iPSC-derived cardiomyocytes have identified that reduced expression of PPARa in immature cardiac cells impedes cell maturation [58]. Furthermore, treatment with the PPARa agonist fenofibrate promoted cardiomyocyte cell maturation [58, 59]. Alternatively, in a murine model of acute myocardial infarction studies determined that treatment of cardiomyocytes with the RXR agonist bexarotene [60] improved cardiomyocyte proliferation and survival. Potentially fenofibrate or bexarotene may ameliorate ongoing cardiomyocyte dysfunction that persists long after CHD surgical intervention.

Currently, due to improved surgical interventions, CHD mortality rates in infancy are declining with over 80% of patients now surviving to adulthood, and the interest in the burden of disease has shifted to include adults (> 65years) with disease-associated, high mortality rates [5]. An understanding of the genetic basis of CHD is becoming increasingly important where early interventions and ongoing disease management may improve life outcomes. The use of a patient-specific precision medicine, through gene editing and human cardiac disease modelling, provides an informative approach to diagnosis and treatment. Genetic variants can be assessed in these models to deliver rapid and accurate VUS interpretation to elucidate disease aetiology, identify key cellular changes, and inform patient-specific treatments. The potential to identify treatments that ameliorate cardiac cell dysfunction and promote the formation of healthy cardiomyocytes may reduce mortality rates in the ageing CHD demographic.

The study demonstrates iPSC gene editing and cardiomyocyte differentiation as a model for understanding CHD in a patient-specific manner. The *GATA4* genetic variant cardiomyocytes were able to recapitulate the patient phenotype and identify changes in cell function and pathways that contribute to disease pathogenesis. Specifically, GATA4 VUS expression in cardiomyocytes revealed disease mechanisms consisting of dysfunction in cardiogenesis, cardiomyocyte contractile strength, calcium handling, and energy metabolism.

Limitations of the study include the use of 2D cultures to model cardiac disease, and further information may be provided by the use of cardiac organoids or further maturation of cardiomyocytes. Developments in the cardiac organoids may enable better recapitulate of CHD with the organisation of heart-like tissues [61, 62]. In addition, studies report iPSC derived cardiomyocytes are often of an immature phenotype, with mechanical load, electrical stimulation and regulation of energy metabolism as potential stimuli to induce the formation of mature cardiomyocytes [63].

The study data provides supporting evidence toward the pathogenicity of the GATA4 c.851G > A p.Arg284His genetic variant in functional in vivo analysis. Further investigation of the family, subsequent to these findings, has identified the GATA4 variant in the probands father with ASD; and, the probands sister with VSD and mitral valve stenosis and her daughter with VSD.

The research study highlights the use of genetically modified iPSC-derived to investigate the impact of patient-specific genetic variants, that are rare or ultra-rare. Recent advances in CRISPR gene editing efficiency in iPSCs [18, 19, 21, 22] for genetic variant introduction enable the rapid selection of clonal cell lines. Furthermore, the use of Cas9 ribonucleoprotein complexes, with high purity crRNA and ssDNA repair strands, improves gene editing accuracy and reduces off-target effects. The capacity for iPSCs to be differentiated to many cell types enables genetic variant induced functional changes to be investigated in a disease relevant setting, in cells that are otherwise of normal karyotype. In summary, advances in CRISPR gene editing in iPSCs enable the rapid generation of patient-specific disease models for genetic variant interpretation.

## Supplementary Information

Supplementary Material 1

Supplementary Material 2

Supplementary Material 3

Supplementary Material 4

Supplementary Material 5

## Data Availability

These are datasets are grouped as two different GEO datasets: **GSE305730** for RNASeq, and **GSE305687** for AmpSeq. Sanger sequencing files can be found at [https://zenodo.org/records/16899266](https:/zenodo.org/records/16899266). All code required to reproduce analysis and figures is provided in The Supplemental Experimental Procedures (Paper Analysis section).
