# Supplementary material for: A precision medicine approach to interpret a GATA4 genetic variant in a paediatric patient with congenital heart disease
Source: Hum Genomics. 2026 Jan 3;20:29. doi: 10.1186/s40246-025-00907-6 (PMC12866568; doi:10.1186/s40246-025-00907-6)
Supplement: Supplementary file 1 — Supplementary Material 1 [file 40246_2025_907_MOESM1_ESM.pdf]

GATA4 VUS   CRISPRESSO Script

```
docker run -v ${PWD}:/DATA -w /DATA pinellolab/crispresso2:v2.0.30
CRISPRessoBatch -bs GATA4VUS2_Batch_Run80.txt -a
cacctttttacttggacatgaagcatttgtttcctgtcttgcagtccgcctcccgccgagtgggcctctcctgtgc
caactgccagaccaccaccaccacgctgtggcgccgcaatgcggagggcgagcctgtgtgcaatgcctgcggcct
ctacatgaagctccacggggtacgtgggtcctgcgcccatgcggcatccttgccttctgatgcccatctctcagt
cctcccttgtcttcttcctttgtac -g cacgctgtggcgccgcaatg -e
cacctttttacttggacatgaagcatttgtttcctgtcttgcagtccgcctcccgccgagtgggcctctcctgtgc
caactgccagaccaccaccaccacgctgtggcgccacaatgcggagggcgagcctgtgtgcaatgcctgcggcct
ctacatgaagctccacggggtacgtgggtcctgcgcccatgcggcatccttgccttctgatgcccatctctcagt
cctcccttgtcttcttcctttgtac --min_paired_end_reads_overlap 10 --
max_paired_end_reads_overlap 10 -w 30 --plot_window_size 30 --
min_frequency_alleles_around_cut_to_plot 0.1
```

GATA4 VUS2 – supplementary code

Kevin Chen

2025-06-06

Table of contents

1 Introduction and preparation **1**

2 Create edgeR `DGEList` and annotate genes **3**

3 Filtering and normalisation **4**
3.1 Library-size and density QC 4
3.2 Normalisation 5
3.3 PCA (MDS) on normalised counts 6

4 Preparing visualisation of expression **7**
4.1 Gene Expression Boxplots 8

5 Differential gene expression **12**

6 Gene-set enrichment analyses **15**
6.1 Dotplot Generation Code 15

7 KEGG Pathway Visualization **18**
7.1 KEGG pathway hsa03320 19

8 Session Info **19**

1 Introduction and preparation

This documenmt provides the code used to analyse results and generate figures for the exploration of the GATA4 VUS2 variant. This involves a routine RNAseq analysis pipeline starting from Kallisto quantifications, with differential gene expression analysis using a standardised **limma** and **edgeR** pipeline, and additional gene set enrichment analyses. Begin by loading libraries and preparing other necessary objects for analysis, including metadata and Kallisto quantification paths.

```r
library(pacman)
p_load(
  tidyverse, tximport, limma, edgeR,
  org.Hs.eg.db, DOSE, enrichplot,
  patchwork, clusterProfiler, viridis, matrixStats,
  pathview
)

metadata <- read_csv("./input_data/GATA4_VUS2_metadata.csv")
```

```
Rows: 16 Columns: 10
-- Column specification --------------------------------------------------------
Delimiter: ","
chr (4): cell_type, cell_ID, experiment_name, GWA_label
dbl (6): sample, experiment_number, paired_samples, ng/ul, 260/280, 260/230

i Use `spec()` to retrieve the full column specification for this data.
i Specify the column types or set `show_col_types = FALSE` to quiet this message.
```

```r
paths <- list.files(
  pattern = "abundance.h5", full.names = TRUE,
  recursive = TRUE
)
paths <- data.frame(
  path = paths,
  GWA_label = str_remove_all(
    paths, "./input_data/Kallisto/|/abundance.h5"
  )
)
paths$GWA_label <- str_replace_all(paths$GWA_label, "-", "_")

metadata.new <- full_join(metadata, paths, by = "GWA_label") %>%
  mutate(
    paired_samples = as.character(paired_samples),
    genotype = if_else(str_detect(GWA_label, "HDR"), "VUS2", "WT"),
    merged_group = paste0(genotype, "_", cell_type)
  )

tx2gene <- read_csv("./input_data/tx2gene_entrez_v38.csv", col_names = FALSE)
```

```
Rows: 201291 Columns: 2
-- Column specification --------------------------------------------------------
Delimiter: ","
chr (1): X1
```

```
dbl (1): X2

i Use `spec()` to retrieve the full column specification for this data.
i Specify the column types or set `show_col_types = FALSE` to quiet this message.
```

```
kallisto <- tximport(metadata.new$path,
  type = "kallisto",
  tx2gene = tx2gene,
  countsFromAbundance = "lengthScaledTPM",
  ignoreAfterBar = TRUE
)
```

```
1 2 3 4 5 6 7 8 9 10 11 12 13 14 15 16
removing duplicated transcript rows from tx2gene
transcripts missing from tx2gene: 63561
summarizing abundance
summarizing counts
summarizing length
summarizing inferential replicates
```

2 Create edgeR `DGEList` and annotate genes

Prepared objects/counts are used to generate the `DGEList` object, which is the main data structure used in **edgeR** for RNA-seq analysis. We also annotate the genes with their Entrez IDs and symbols using the `bitr` function from the **clusterProfiler** package to facilitate downstream analyses.

```
DGE <- DGEList(kallisto$counts)
DGE$samples <- bind_cols(DGE$samples, metadata.new)
DGE$genes <- bitr(
  geneID = rownames(DGE), fromType = "ENTREZID",
  toType = "SYMBOL", OrgDb = "org.Hs.eg.db", drop = FALSE
)
```

```
'select()' returned 1:1 mapping between keys and columns

Warning in bitr(geneID = rownames(DGE), fromType = "ENTREZID", toType =
"SYMBOL", : 0.08% of input gene IDs are fail to map...
```

```
colnames(DGE$genes) <- c("GeneID", "Symbol")
identical(rownames(DGE), DGE$genes$GeneID) # sanity check
```

```
[1] TRUE
```

3 Filtering and normalisation

Filter out lowly expressed genes and perform TMM normalisation. Visualise the results as we go to assess the quality of the data.

```
design <- model.matrix(~ 0 + paired_samples, data = DGE$samples)

genotypes <- unique(DGE$samples$genotype)
template.matrix <- matrix(0, nrow = ncol(DGE), ncol = length(genotypes))
colnames(template.matrix) <- genotypes

define_design_matrix <- function(x) {
  meta <- DGE$samples %>% select(genotype, cell_type)
  if (meta$cell_type[x] == "CM") {
    template.matrix[x, which(genotypes == meta$genotype[x])] <- 1
  }
  template.matrix[x, ]
}
final.matrix <- t(sapply(1:ncol(DGE), define_design_matrix))
design.paired <- cbind(design, final.matrix)

keep.exprs <- filterByExpr(DGE, design = design.paired)
keep.exprs[is.na(DGE$genes$Symbol)] <- FALSE
DGE.filtered <- DGE[keep.exprs, keep.lib.sizes = FALSE]
```

3.1 Library-size and density QC

We assess the logCPM distribution of the unfiltered and filtered data. The unfiltered data contains many genes with low expression (large spike on the left where logCPM = -5). This is largely removed with the filtered data, demonstrating the filtering was able to remove these lowly expressed genes.

```
L <- mean(DGE$samples$lib.size) * 1e-6
M <- median(DGE$samples$lib.size) * 1e-6
lcpm.cutoff <- log2(10 / M + 2 / L)
nsamples <- ncol(DGE)
col <- inferno(nsamples, direction = -1)

par(mfrow = c(1, 2))
lcpm <- cpm(DGE, log = TRUE)
plot(density(lcpm[, 1]),
  col = col[1], lwd = 2,
  ylim = c(0, 0.2), las = 2, main = "", xlab = ""
)
title(main = "A. Unfiltered data", xlab = "Log-cpm")
```

```
abline(v = lcpm.cutoff, lty = 3)
for (i in 2:nsamples) {
  lines(density(lcpm[, i]), col = col[i], lwd = 2)
}
lcpm <- cpm(DGE.filtered, log = TRUE)
plot(density(lcpm[, 1]),
  col = col[1], lwd = 2,
  ylim = c(0, 0.2), las = 2, main = "", xlab = ""
)
title(main = "B. Filtered data", xlab = "Log-cpm")
abline(v = lcpm.cutoff, lty = 3)
for (i in 2:nsamples) {
  lines(density(lcpm[, i]), col = col[i], lwd = 2)
}
```

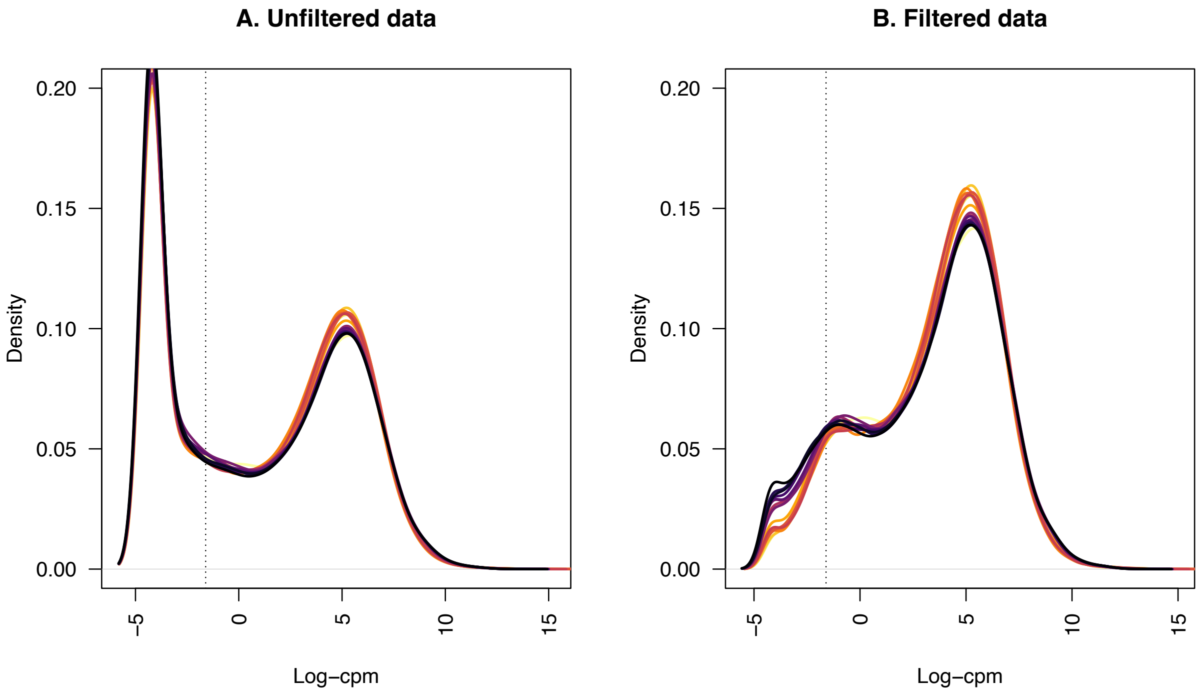

3.2 Normalisation

Trimmed Mean of M-values (TMM) normalisation is applied to account for compositional biases between samples. The boxplots show the distribution of log-CPM values before and after normalisation, with the normalised boxplots being slightly more evenly distributed across samples.

```
DGE.final <- calcNormFactors(DGE.filtered)
par(mfrow = c(1, 2))
boxplot(cpm(DGE.filtered, log = TRUE), las = 2, main = "")
```

```
title(main = "A. Unnormalised", ylab = "Log-cpm")
boxplot(cpm(DGE.final, log = TRUE), las = 2, main = "")
title(main = "B. Normalised", ylab = "Log-cpm")
```

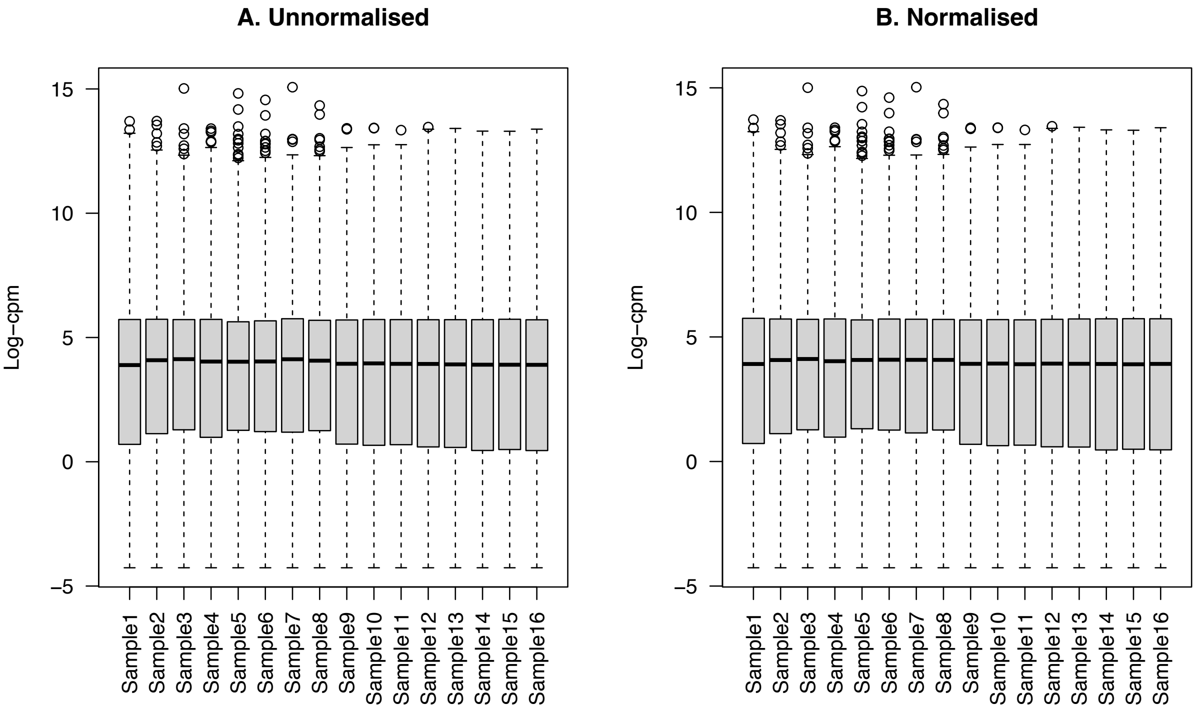

3.3 PCA (MDS) on normalised counts

We visualise the normalised counts using a multidimensional scaling (MDS) plot, which provides an overview of sample relationships based on gene expression profiles. Text labels indicate iPSCs ("Stem") vs. cardiomyocytes ("Cardio"), and demonstrates separation between these.

```
lcpm <- cpm(DGE.final, log = TRUE)
geno_colour <- c("red", "blue")[as.factor(DGE$samples$genotype)]
par(mfrow = c(1, 1))
mds <- plotMDS(lcpm, col = geno_colour, labels = DGE$samples$GWA_label)
```

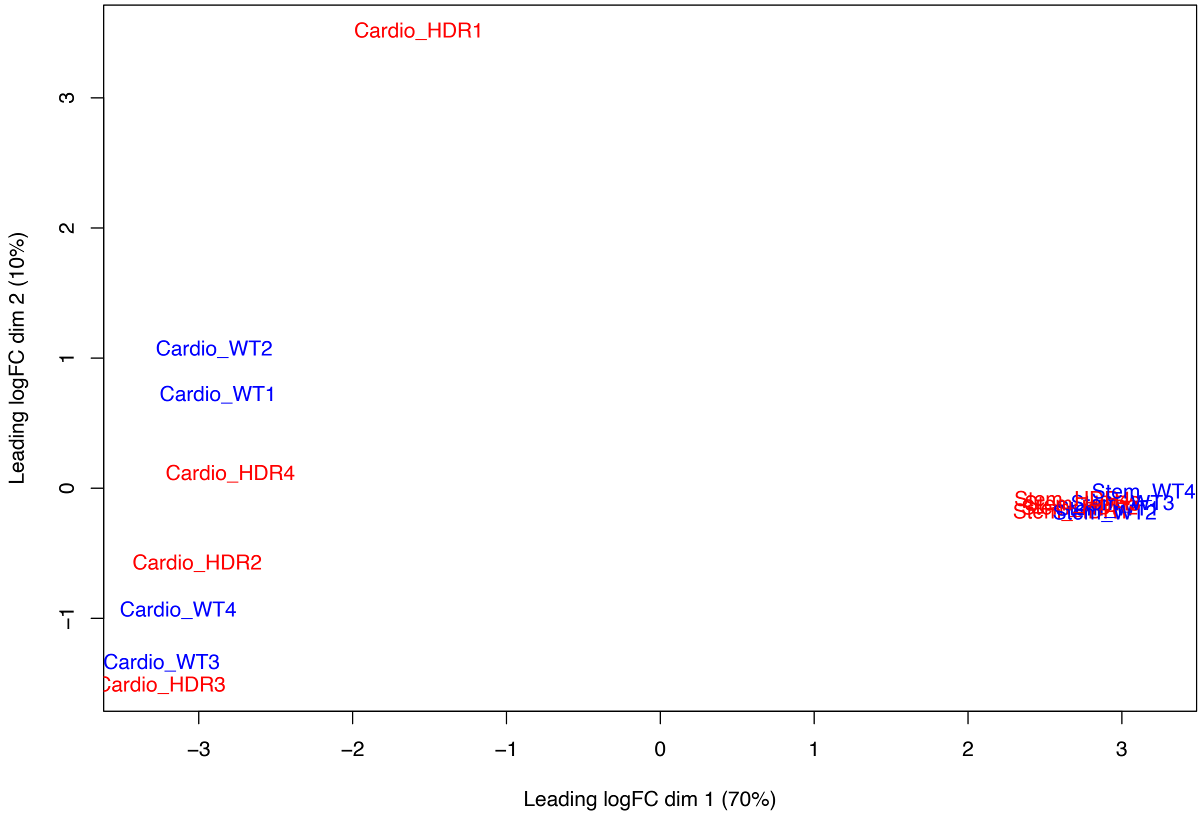

4 Preparing visualisation of expression

We prepare a counts-per-million (CPM) table with gene symbols and identifiers for downstream analysis and export it to a CSV file.

```
cpm_tbl <- cpm(DGE.final, normalized.lib.sizes = FALSE) %>% as.data.frame()
colnames(cpm_tbl) <- DGE.final$samples$GWA_label
cpm_tbl$Symbol <- bitr(rownames(cpm_tbl),
  fromType = "ENTREZID",
  toType = "SYMBOL", OrgDb = "org.Hs.eg.db"
)$SYMBOL
```

```
'select()' returned 1:1 mapping between keys and columns
```

```
cpm_tbl <- cpm_tbl %>%
  rownames_to_column("GeneID") %>%
  select(Symbol, GeneID, everything())
write_csv(cpm_tbl, "./results/GATA4_cpm.csv")
```

4.1 Gene Expression Boxplots

Expression boxplots provide a visualisation of key gene expression patterns across different cell types and genotypes. Here we analyze expression of critical genes including:

- Pluripotency markers (POU5F1/Oct4, NANOG)
- Cardiac markers (TNNT2/cTnT, MYBPC3)
- GATA4 itself (the gene containing our variant of interest)

These plots help us understand how the GATA4 VUS2 variant affects both stem cell and cardiomyocyte gene expression, particularly focusing on genes involved in cardiac development and function.

```
# Generate expression data for key genes
genes <- bitr(
  geneID = c(
    "POU5F1",
    "NANOG",
    "TNNT2",
    "MYBPC3",
    "GATA4"
  ),
  fromType = "SYMBOL",
  toType = "ENTREZID",
  OrgDb = "org.Hs.eg.db"
)
```

```
'select()' returned 1:1 mapping between keys and columns
```

```
geneIDs <- genes$ENTREZID
geneSymbols <- genes$SYMBOL

# Extract CPM values for these genes and format for plotting
cpm_for_plotting <- cpm(DGE.final)[geneIDs, ] %>%
  as.data.frame()
rownames(cpm_for_plotting) <- geneSymbols
colnames(cpm_for_plotting) <- DGE.final$samples$GWA_label

cpm_for_plotting <- cpm_for_plotting %>%
  rownames_to_column(var = "gene") %>%
  pivot_longer(
    cols = matches("^(Cardio|Stem)"),
    names_to = "sample_name",
    values_to = "cpm"
  ) %>%
  mutate(
```

```r
    celltype = factor(
      if_else(str_detect(sample_name, "Cardio"),
        "Cardiomyocyte",
        "iPSC"
      ),
      levels = c("iPSC", "Cardiomyocyte")
    ),
    genotype = factor(
      if_else(str_detect(sample_name, "WT"),
        "WT",
        "VUS2"
      ),
      levels = c("WT", "VUS2")
    ),
    merged_group = paste0(celltype, "_", genotype)
  )

# Create expression boxplot
p <- ggplot(
  data = cpm_for_plotting,
  aes(
    x = genotype,
    y = cpm,
    fill = merged_group
  )
) +
  geom_boxplot(outlier.shape = NULL) +
  geom_point(aes(group = merged_group),
    position = position_dodge(width = 0.75)
  ) +
  facet_grid(
    rows = vars(gene),
    cols = vars(celltype),
    scale = "free_y",
    switch = "both"
  ) +
  xlab(NULL) +
  ylab("Counts per million") +
  scale_fill_manual(
    name = "Type of cell",
    values = c("red", "#4685eb", "pink", "lightblue"),
    labels = c(
      "Cardiomyocyte - VUS2",
      "Cardiomyocyte - WT",
      "iPSC - VUS2",
```

```
      "iPSC - WT"
    )
  ) +
  theme(legend.position = "none")

# Display the plot
print(p)
```

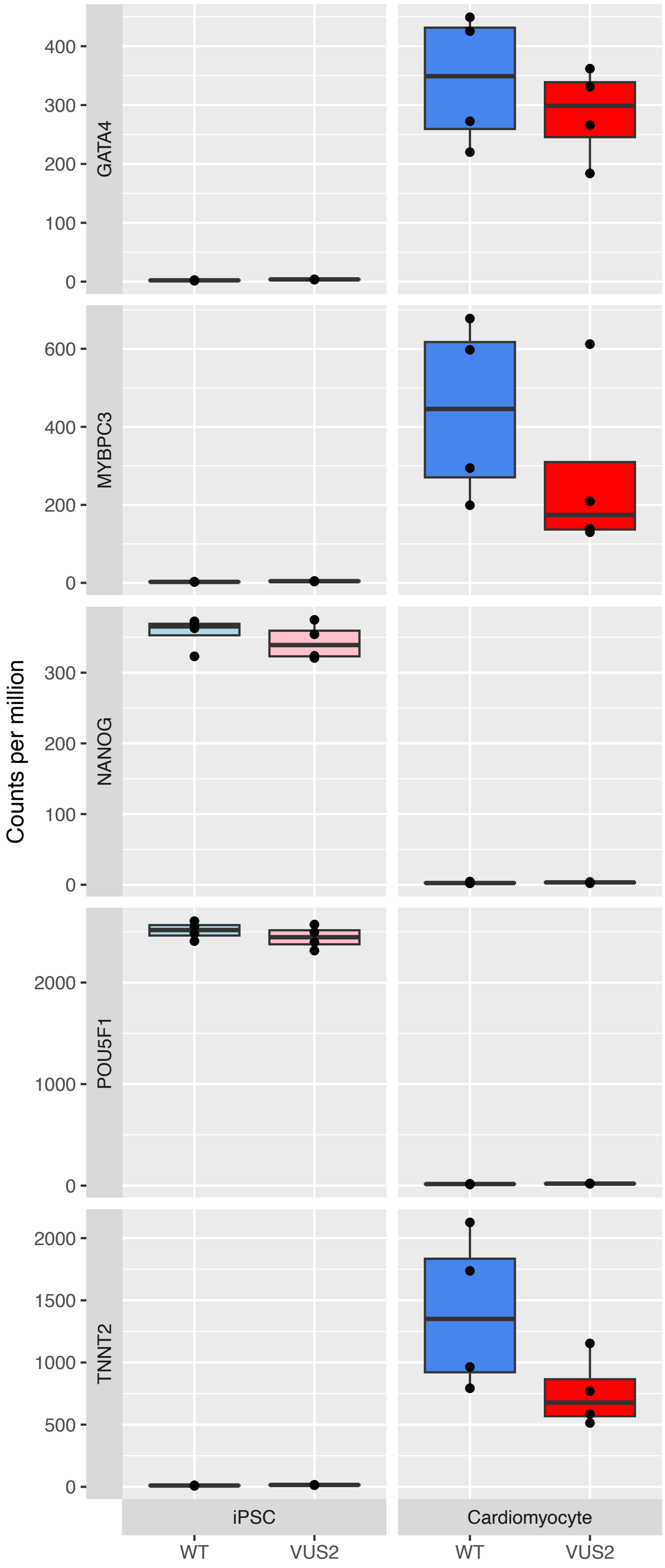

Counts per million
GATA4
MYBPC3
NANOG
POU5F1
TNNT2
iPSC
Cardiomyocyte
WT
VUS2
WT
VUS2
0
100
200
300
400
600
1000
1500
2000
500

5 Differential gene expression

We conduct a standardised differential gene expression (DGE) analysis using the **limma** and **edgeR** packages. This involves fitting linear models to the data, applying empirical Bayes moderation. We assess WT differentiation, VUS2 differentiation, the difference between VUS2 and WT cardiomyocytes, as well as the difference in differentiation betwen the two genotypes. The DGE analysis allows us to identify genes that have expression patterns changed by the GATA4 VUS2 variant, pointing to avenues for further investigation.

```
##   build the unpaired design
design.unpaired <- model.matrix(~ 0 + merged_group,
  data = DGE.final$samples
)
colnames(design.unpaired) <- str_remove_all(
  colnames(design.unpaired),
  "merged_group"
)

contrast.matrix.paired <- makeContrasts(
  WT_differentiation = "WT",
  VUS2_differentiation = "VUS2",
  difference_of_differences = "VUS2 - WT",
  levels = colnames(design.paired)
)

contrast.matrix.unpaired <- makeContrasts(
  difference_between_CMs = VUS2_CM - WT_CM,
  levels = colnames(design.unpaired)
)

##   fit the paired model
v <- voom(DGE.final, design.paired, plot = TRUE)
```

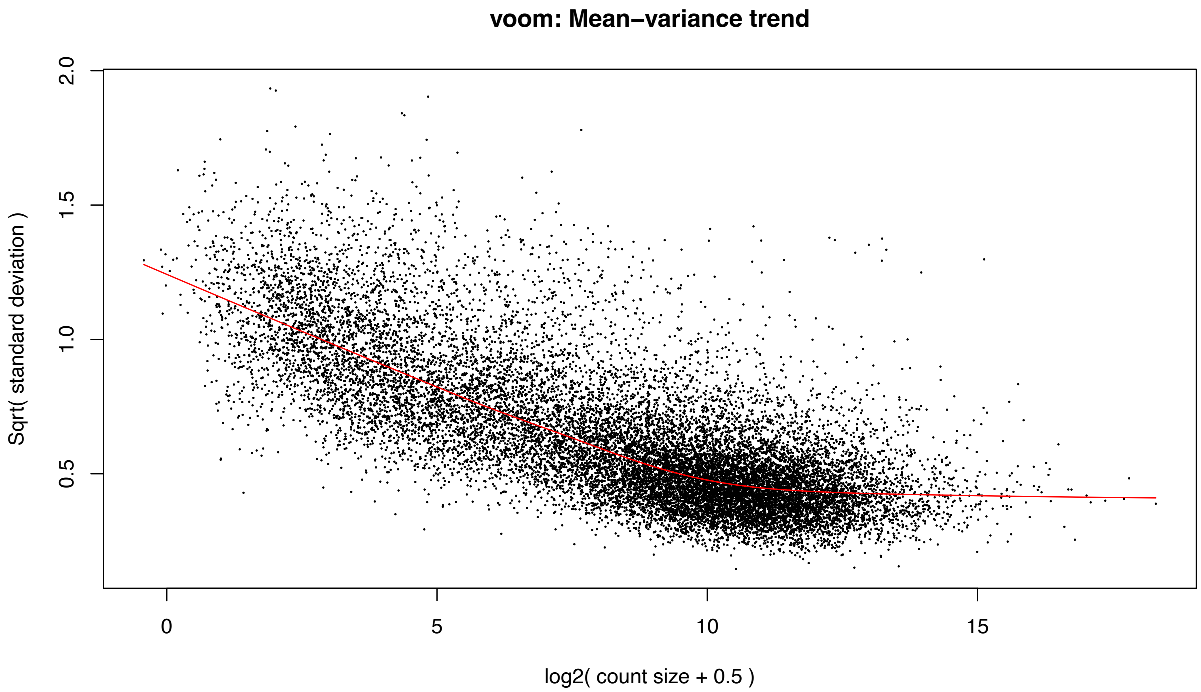


```r
vfit <- lmFit(v, design.paired)
vfit <- contrasts.fit(vfit, contrasts = contrast.matrix.paired)
efit_paired <- eBayes(vfit) # paired result

##   fit the unpaired model
v <- voom(DGE.final, design.unpaired, plot = TRUE)
```

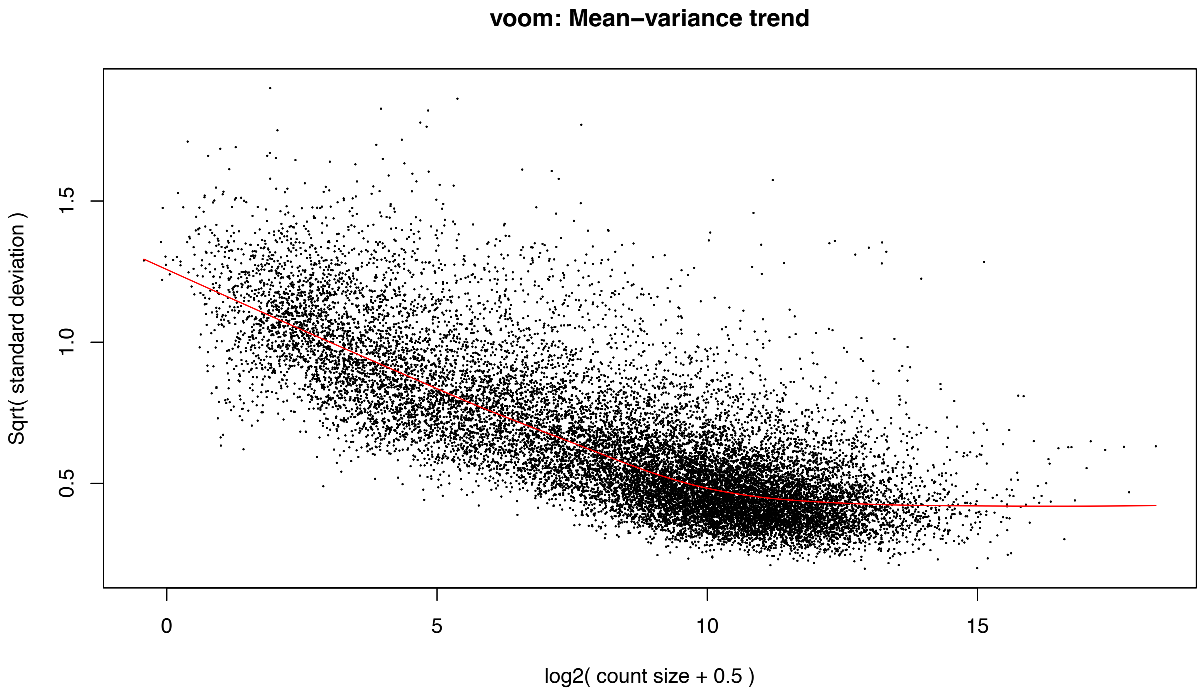

```r
vfit <- lmFit(v, design.unpaired)
vfit <- contrasts.fit(vfit, contrasts = contrast.matrix.unpaired)
efit_unpaired <- eBayes(vfit)

##   create LFC tables and ranked gene lists for GSEA
contrasts.paired <- colnames(contrast.matrix.paired)
contrasts.unpaired <- colnames(contrast.matrix.unpaired)
contrasts <- c(contrasts.paired, contrasts.unpaired)

LFC.summary <- sapply(contrasts, function(x) {
  lfc.list <- list()
  if (x %in% contrasts.paired) {
    top <- topTable(efit_paired,
      coef   = x,
      number = Inf
    ) %>% list()
    lfc.list <- append(lfc.list, top)
  } else {
    top <- topTable(efit_unpaired,
      coef   = x,
      number = Inf
    ) %>% list()
    lfc.list <- append(lfc.list, top)
  }
})

GSEA.lists <- sapply(contrasts, function(x) {
  gsea.list <- list()
  if (x %in% contrasts.paired) {
    top <- topTable(efit_paired,
      coef   = x,
      number = Inf
    ) %>%
      arrange(desc(logFC))
    lfc <- top$logFC
    names(lfc) <- top$GeneID
    gsea.list <- append(gsea.list, list(lfc))
  } else {
    top <- topTable(efit_unpaired,
      coef   = x,
      number = Inf
    ) %>%
      arrange(desc(logFC))
    lfc <- top$logFC
    names(lfc) <- top$GeneID
```

```
    gsea.list <- append(gsea.list, list(lfc))
  }
})
```

6 Gene-set enrichment analyses

We run gene set enrichment analyses (GSEA) using the **clusterProfiler** package. Specifically, this performs a preranked GSEA, and we use the log fold change list (as generated from the DGE steps) for all contrasts. The GSEA allows us to identify pathways that are affected by the GATA4 VUS2 variant, providing insights into the biological processes and pathways that may be disrupted. The code uses DisGeNET as an example, though the same analysis is performed on other datasets as well (GO, KEGG). The code for these additional datasets is identical, with the only difference being the `fun` argument in the `compareCluster` function.

```
set.seed(42)

DGN.p005 <- compareCluster(
  geneClusters  = GSEA.lists,
  fun           = "gseDGN",
  pvalueCutoff  = 0.05,
  pAdjustMethod = "BH",
  nPermSimple   = 10000,
  seed          = 42,
  eps           = 0
) |>
  setReadable("org.Hs.eg.db", keyType = "ENTREZID")
```

6.1 Dotplot Generation Code

The following code was used to generate dotplots for gene set enrichment analysis results. This creates standardised visualisations of enriched terms from DisGeNET and other ontologies. The dotplots show enriched disease terms with dot size representing gene ratio and colour indicating normalised enrichment score (NES).

```
# Load required objects (if not already loaded)
DGN_ck <- readRDS("./scratch/R_objects/DGN_unfilered.RDS")
terms <- read_csv("./input_data/2024-02-20_DotplotTerms/CollatedInputTerms.csv") %>%
  dplyr::select(1:4)
```

```
New names:
Rows: 60 Columns: 5
-- Column specification
```

```
---------------------------------------------------------- Delimiter: "," chr
(4): Contrast, ONTOLOGY, ID, Description lgl (1): ...5
i Use `spec()` to retrieve the full column specification for this data. i
Specify the column types or set `show_col_types = FALSE` to quiet this message.
* `` -> `...5`
```

```
# Generic function for creating dotplots
CreateDotplot <- function(ck_object,
                          ontology,
                          output_name,
                          height = 8,
                          width = 14) {
  plot_terms <- terms %>%
    filter(ONTOLOGY == ontology) %>%
    pull(Description) %>%
    unique()

  temp <- ck_object
  temp@compareClusterResult <- temp@compareClusterResult %>%
    filter(p.adjust < 0.05 &
      Cluster %in% c("difference_of_differences", "difference_between_CMs")) %>%
    mutate(Cluster = factor(Cluster,
      level = c(
        "difference_between_CMs",
        "difference_of_differences"
      )
    ))

  p <- dotplot(temp,
    showCategory = plot_terms,
    color = "NES",
    font.size = 8,
    label_format = 100
  ) +
    scale_fill_gradient2(
      low = "blue",
      mid = "white",
      high = "red"
    ) +
    scale_x_discrete(labels = c(
      "Difference \n between \n cardiomyocytes",
      "Difference of \n differences"
    )) +
    xlab(NULL) +
    theme(
      axis.text.x = element_text(
```

```
        angle = 0,
        hjust = 0.5,
        size = 8
      ),
      axis.text.y = element_text(size = 8),
      plot.margin = unit(
        c(0.5, -0.5, 0.5, 0.5),
        "cm"
      )
    )

  p
}

# Create dotplots for different ontologies - example for DisGeNET
CreateDotplot(
  ck_object = DGN_ck,
  ontology = "DGN",
  output_name = "DisGeNET"
)
```

```
Scale for fill is already present.
Adding another scale for fill, which will replace the existing scale.
```

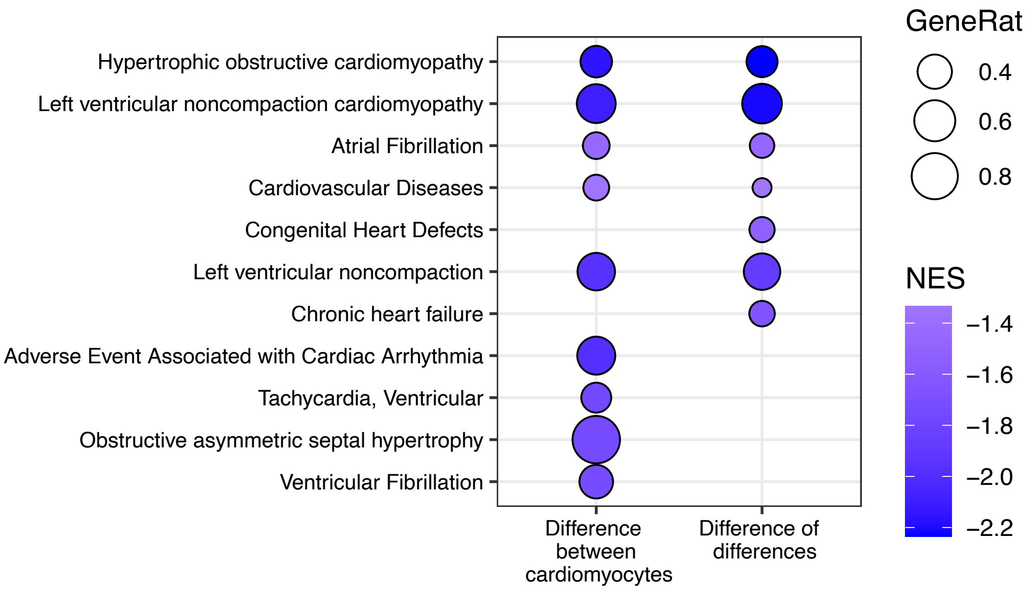

7 KEGG Pathway Visualization

KEGG pathway visualization using the `pathview` package allows us to map our differential expression results onto known biological pathways. This provides a systems-level view of how the GATA4 VUS2 variant affects cellular processes. We visualize several key pathways relevant to cardiac development and function, though code is only shown for one pathway (PPAR signaling pathway, hsa03320) as an example. Code for other pathways is similar, with the only difference being the `pathway.id` argument in the `pathview` function.

```
# Use the difference_between_CMs contrast for pathway visualization
genelist <- GSEA.lists[["difference_between_CMs"]]

# PPAR signaling pathway
pathview(
  gene.data = genelist,
  pathway.id = "hsa03320",
  species = "hsa",
  low = list(gene = "steelblue"),
  limit = 2
)
```

```
'select()' returned 1:1 mapping between keys and columns

Info: Working in directory /Users/kevinchen/Documents/rnaseq/GATA4_VUS2

Info: Writing image file hsa03320.pathview.png
```

7.1 KEGG pathway hsa03320

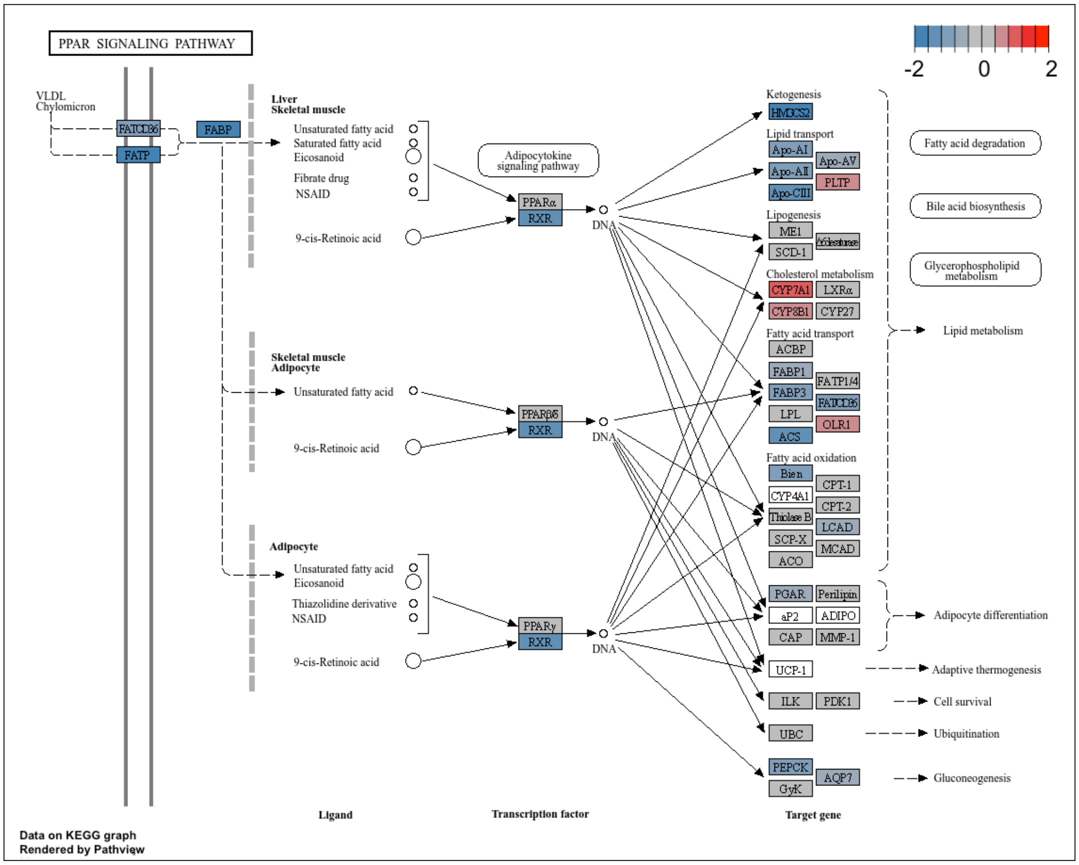

Figure 1: Pathview rendering of the PPAR signaling pathway (hsa03320). Colour represents the log fold change of genes in their respective node, as indicated in the legend in the top right - where a node represents multiple genes, the mean log fold change is used. Blue indicates downregulation, red indicates upregulation.

8 Session Info

```r
sessionInfo()
```

```
R version 4.4.3 (2025-02-28)
Platform: aarch64-apple-darwin23.6.0
Running under: macOS Sonoma 14.5

Matrix products: default
BLAS:   /opt/homebrew/Cellar/openblas/0.3.29/lib/libopenblasp-r0.3.29.dylib
LAPACK: /opt/homebrew/Cellar/r/4.4.3_1/lib/R/lib/libRlapack.dylib;  LAPACK version 3.12.0
```

```
locale:
[1] en_US.UTF-8/en_US.UTF-8/en_US.UTF-8/C/en_US.UTF-8/en_US.UTF-8

time zone: Australia/Perth
tzcode source: internal

attached base packages:
[1] stats4    stats     graphics  grDevices utils     datasets  methods
[8] base

other attached packages:
 [1] pathview_1.44.0        matrixStats_1.3.0      viridis_0.6.5
 [4] viridisLite_0.4.2      clusterProfiler_4.12.2 patchwork_1.2.0
 [7] enrichplot_1.24.2      DOSE_3.30.2            org.Hs.eg.db_3.19.1
[10] AnnotationDbi_1.66.0   IRanges_2.38.1         S4Vectors_0.42.1
[13] Biobase_2.64.0         BiocGenerics_0.50.0    edgeR_4.2.1
[16] limma_3.60.4           tximport_1.32.0        lubridate_1.9.3
[19] forcats_1.0.0          stringr_1.5.1          dplyr_1.1.4
[22] purrr_1.0.2            readr_2.1.5            tidyr_1.3.1
[25] tibble_3.2.1           ggplot2_3.5.1          tidyverse_2.0.0
[28] pacman_0.5.1

loaded via a namespace (and not attached):
  [1] RColorBrewer_1.1-3      jsonlite_1.8.8          magrittr_2.0.3
  [4] farver_2.1.2            rmarkdown_2.27          fs_1.6.4
  [7] zlibbioc_1.50.0         vctrs_0.6.5             memoise_2.0.1
 [10] RCurl_1.98-1.16         ggtree_3.12.0           tinytex_0.52
 [13] htmltools_0.5.8.1       Rhdf5lib_1.26.0         rhdf5_2.48.0
 [16] gridGraphics_0.5-1      plyr_1.8.9              cachem_1.1.0
 [19] igraph_2.0.3            lifecycle_1.0.4         pkgconfig_2.0.3
 [22] Matrix_1.7-0            R6_2.5.1                fastmap_1.2.0
 [25] gson_0.1.0              GenomeInfoDbData_1.2.12 digest_0.6.36
 [28] aplot_0.2.3             colorspace_2.1-1        RSQLite_2.3.7
 [31] labeling_0.4.3          fansi_1.0.6             timechange_0.3.0
 [34] httr_1.4.7              polyclip_1.10-7         compiler_4.4.3
 [37] bit64_4.0.5             withr_3.0.0             BiocParallel_1.38.0
 [40] DBI_1.2.3               ggforce_0.4.2           MASS_7.3-64
 [43] HDO.db_0.99.1           tools_4.4.3             ape_5.8
 [46] scatterpie_0.2.3        glue_1.7.0              rhdf5filters_1.16.0
 [49] nlme_3.1-167            GOSemSim_2.30.0         grid_4.4.3
 [52] shadowtext_0.1.4        reshape2_1.4.4          fgsea_1.30.0
 [55] generics_0.1.3          gtable_0.3.5            tzdb_0.4.0
 [58] data.table_1.15.4       hms_1.1.3               tidygraph_1.3.1
 [61] utf8_1.2.4              XVector_0.44.0          ggrepel_0.9.5
 [64] pillar_1.9.0            vroom_1.6.5             yulab.utils_0.1.5
 [67] splines_4.4.3           tweenr_2.0.3            treeio_1.28.0
```

```
 [70] lattice_0.22-6          bit_4.0.5               tidyselect_1.2.1
 [73] GO.db_3.19.1            locfit_1.5-9.10         Biostrings_2.72.1
 [76] knitr_1.48              gridExtra_2.3           xfun_0.46
 [79] graphlayouts_1.1.1      statmod_1.5.0           KEGGgraph_1.64.0
 [82] stringi_1.8.4           UCSC.utils_1.0.0        lazyeval_0.2.2
 [85] ggfun_0.1.5             yaml_2.3.10             evaluate_0.24.0
 [88] codetools_0.2-20        ggraph_2.2.1            qvalue_2.36.0
 [91] Rgraphviz_2.48.0        graph_1.82.0            ggplotify_0.1.2
 [94] cli_3.6.3               munsell_0.5.1           Rcpp_1.0.13
 [97] GenomeInfoDb_1.40.1     png_0.1-8               XML_3.99-0.17
[100] parallel_4.4.3          blob_1.2.4              bitops_1.0-8
[103] tidytree_0.4.6          scales_1.3.0            crayon_1.5.3
[106] rlang_1.1.4             cowplot_1.1.3           fastmatch_1.1-4
[109] KEGGREST_1.44.1
```
