# Supplementary material for: A precision medicine approach to interpret a GATA4 genetic variant in a paediatric patient with congenital heart disease
Source: Hum Genomics. 2026 Jan 3;20:29. doi: 10.1186/s40246-025-00907-6 (PMC12866568; doi:10.1186/s40246-025-00907-6)
Supplement: Supplementary file 4 — Supplementary Material 4 [file 40246_2025_907_MOESM4_ESM.docx]

**ADDITIONAL INFORMATION**

Supplementary Figures

**Suppl Figure 1**. **Quality control on GATA4 VUS and matched GATA4 WT clonal cell lines.** **A,** Representative sanger sequencing at off target sites. **B,** Karyotyping analysis report indicates same chr20q amplification across all clones, and the parental KOLF2 cell line.

**Suppl Figure 2. Transcriptomics identifies changes during cardiomyocyte differentiation of GATA4 WT and GATA4 VUS cells. A,** Principal component analysis UMAP indicating separation of iPSCs from cardiomyocytes. Cardio_WT indicates GATA4 WT cells, and Cardio_HDR2 indicates GATA4 VUS cells. **B,** Transcriptomics analysis revealed changes in stem and C, cardiac cell markers, during cardiomyocyte differentiation, box plots as indicated. (log_2_fold change; p adj <0.05; n=4).

**Suppl Figure 3. Transcriptomics identifies changes in gene expression for GATA4 WT and GATA4 VUS cardiomyocytes.** Differentially expressed genes in the comparison of GATA4 VUS to GATA4 WT cardiomyocytes.

**Suppl Figure 4. Changes in calcium pathways in GATA4 VUS genetic variant cardiomyocytes.** Transcriptomics analysis with GSEA and KEGG database comparing GATA4 VUS to GATA4 WT for the differences between cardiomyocytes, pathway changes in PPAR signalling pathway and Oxidative phosphorylation, as indicated.

Supplementary Tables

**Suppl Table 1.** GATA4 crRNA OT primers

**Suppl Table 2.** Differentially expressed genes in the comparison of GATA4 WT cardiomyocytes to GATA4 WT iPSCs.

**Suppl Table 3.** Differentially expressed genes in the comparison of GATA4 VUS cardiomyocytes to GATA4 VUS iPSCs.

**Suppl Table 4.** GSEA analysis in GO-BP database for the comparison of GATA4 WT cardiomyocytes to GATA4 WT iPSCs.

**Suppl Table 5.** GSEA analysis in GO-BP database for the comparison of GATA4 VUS cardiomyocytes to GATA4 VUS iPSCs.

**Suppl Table 6.** GSEA analysis in DisGeNET database for the comparison of GATA4 VUS cardiomyocytes to GATA4 WT CMs.

**Suppl Table 7.** GSEA analysis in DisGeNET database for the difference in cardiomyocyte differentiation when comparing GATA4 VUS CMs to GATA4 WT.

**Suppl Table 8.** DEG in the comparison of GATA4 VUS to GATA4 WT cardiomyocytes.

**Suppl Table 9.** GSEA analysis in GO-BP database for the comparison of GATA4 VUS to GATA4 WT cardiomyocytes.

**Suppl Table 10.** GSEA analysis in GO-BP database for the difference in cardiomyocyte differentiation comparing GATA4 VUS to GATA4 WT.

**Suppl Table 11.** GSEA analysis in GO-MF database for the comparison of GATA4 VUS to GATA4 WT cardiomyocytes.

**Suppl Table 12.** GSEA analysis in GO-MF database for the difference in cardiomyocyte differentiation comparing GATA4 VUS to GATA4 WT.

**Suppl Table 13.** GSEA analysis in GO-CC database for the comparison of GATA4 VUS to GATA4 WT cardiomyocytes.

**Suppl Table 14.** GSEA analysis in GO-CC database for the difference in cardiomyocyte differentiation comparing GATA4 VUS to GATA4 WT.

**Suppl Table 15.** GSEA analysis in KEGG database for the comparison of GATA4 VUS to GATA4 WT cardiomyocytes.

Supplementary Videos

**Suppl Video 1.** GATA4 WT clone 4.9. Video recording of beating cardiomyocytes.

**Suppl Video 2.** GATA4 VUS clone 4.5. Video recording of beating cardiomyocytes.
